# MicroRNA-194 regulates parasitic load and IL-1β-dependent nitric oxide production in the peripheral blood mononuclear cells of dogs with leishmaniasis

**DOI:** 10.1371/journal.pntd.0011789

**Published:** 2024-01-19

**Authors:** Sidnei Ferro Costa, Matheus Fujimura Soares, Jaqueline Poleto Bragato, Marilene Oliveira dos Santos, Gabriela Torres Rebech, Jéssica Henrique de Freitas, Valéria Marçal Felix de Lima

**Affiliations:** Department of Clinical Medicine, Surgery and Animal Reproduction, São Paulo State University (UNESP), School of Veterinary Medicine, Araçatuba, São Paulo, Brazil; CSIR-Indian Institute of Chemical Biology, INDIA

## Abstract

Domestic dogs are the primary urban reservoirs of *Leishmania infantum*, the causative agent of visceral leishmaniasis. In Canine Leishmaniasis (CanL), modulation of the host’s immune response may be associated with the expression of small non-coding RNAs called microRNA (miR). miR-194 expression increases in peripheral blood mononuclear cells (PBMCs) of dogs with leishmaniasis with a positive correlation with the parasite load and in silico analysis demonstrated that the TRAF6 gene is the target of miR-194 in PBMCs from diseased dogs. Here, we isolated PBMCs from 5 healthy dogs and 28 dogs with leishmaniasis, naturally infected with *L*. *infantum*. To confirm changes in miR-194 and TRAF6 expression, basal expression of miR-194 and gene expression of TRAF6 was measured using qPCR. PBMCs from healthy dogs and dogs with leishmaniasis were transfected with miR-194 scramble, mimic, and inhibitor and cultured at 37° C, 5% CO_2_ for 48 hours. The expression of possible targets was measured: iNOS, NO, T-bet, GATA3, and FoxP3 were measured using flow cytometry; the production of cytokines IL-1β, IL-4, IL-6, IL-10, TNF-α, IFN-γ, and TGF-β in cell culture supernatants was measured using capture enzyme-linked immunosorbent assays (ELISA). Parasite load was measured using cytometry and qPCR. Functional assays followed by miR-194 inhibitor and IL-1β blockade and assessment of NO production were also performed. Basal miR-194 expression was increased in PBMC from dogs with Leishmaniasis and was negatively correlated with TRAF6 expression. The mimic of miR-194 promoted an increase in parasite load. There were no significant changes in T-bet, GATA3, or FoxP3 expression with miR-194 enhancement or inhibition. Inhibition of miR-194 increased IL-1β and NO in PBMCs from diseased dogs, and blockade of IL-1β following miR-194 inhibition decreased NO levels. These findings suggest that miR-194 is upregulated in PBMCs from dogs with leishmaniasis and increases parasite load, possibly decreasing NO production via IL-1β. These results increase our understanding of the mechanisms of evasion of the immune response by the parasite and the identification of possible therapeutic targets.

## Author summary

Visceral leishmaniasis (VL) is a zoonosis in tropical and subtropical regions. In the new world, it is caused by the obligate intracellular protozoan *Leishmania infantum*. In urban areas, domestic dogs are the primary reservoir. Increase in canine leishmaniasis (CanL) contributes to the increase in VL in humans. One mechanism of parasite survival is through regulation of microRNA (miRNA), responsible for translational control of transcripts related to anti-parasite immunity. One example is miR-194, which is increased in the peripheral blood mononuclear cells (PBMCs) of dogs with leishmaniasis. In the present study, we found that increasing miR-194 increases parasite load, and decreasing miR-194 increases IL1β and NO. Subsequently, we found that the increase in NO depends on IL1β. Since NO its directly involved in parasite load control, these findings contribute to understanding how the parasite evades immune responses and paves the way for the discovery of new therapeutic targets.

## Introduction

Visceral Leishmaniasis (VL) is a zoonosis caused in the new world by the obligate intracellular protozoan *Leishmania infantum* (syn. *Leishmania chagasi*) [1]. VL is one of the primary parasitic diseases with the potential for outbreaks and death worldwide; it occurs primarily in tropical and subtropical regions, with an estimated 50.000 to 90.000 new cases annually [2]. Brazil is an endemic region for VL, with 97% of the cases registered in the Americas [2].

The domestic dog is the primary urban reservoir [3]; in endemic regions, there is a correlation between seropositive dogs and the number of cases in humans [4,5]. In canine leishmaniasis (CanL), infection-resistant dogs develop a cellular immune response (Th1) with the production of cytokines IL-2, IL-12, and IFN-γ [6,7] stimulating production of nitric oxide (NO) in infected macrophages and parasite death [8]. By contrast, dogs susceptible to the disease develop a humoral immune response (Th2) with high antibody titers [6] and production of IL-4 and IL-10 cytokines, that inhibit activation of leishmanicidal mechanisms, resulting in increased parasite loads [9].

Modulation of immune response in favor of the parasite in CanL may be associated with the differential expression of microRNAs (miRNAs), which are small non-coding RNAs that bind to their target messenger RNA via base complementarity, resulting in the regulation of translation by messenger RNA degradation [10,11]. In *Leishmania* spp. infections, parasites have developed the ability to exploit the expression of microRNAs and manipulate the phenotype of infected cells to evade the host’s immune response [12].

In this context, in experimental models of *Leishmania donovani* infection, the parasite negatively regulates miRNA expression in murine macrophages through the gp63 surface glycoprotein, a Zinc-metalloprotease that cleaves the Dicer1 protein which is necessary for mature miRNA biogenesis [13]. In human macrophages, the parasite induces increased expression of the transcription factor c-Myc and negatively regulates a group of 19 miRNAs at the level of transcription of the miRNA gene, decreasing the synthesis of primary miRNAs in infected cells [12]. There are still few studies on the mechanisms used by intracellular pathogens to induce changes in the expression of miRNAs in host cells.

In CanL, differential expression of several miRNAs, miR-21, miR-148a, and miR-615 in splenic leukocytes and of miR-21, miR-159, miR-451, miR-192, miR-194, and miR371 in peripheral blood mononuclear cells (PBMCs) can impact immunity-related targets [14,15]. In PBMCs from dogs with leishmaniasis, miR-194 is increased and positively correlates with parasite load [15]. In human diploid fibroblasts and human colorectal carcinoma cell line, miR-194 can be up-regulated by the tumor suppressor p53, which regulates the expression of microRNAs at different levels, p53 might enhance the cleavage processivity of Drosha [16,17] and thus, p53 can promote the processing of specific pri-miRNAs to pre-miRNAs of several microRNAs [18], however, whether the same mechanisms occur in PBMCs from dogs with leishmaniasis still needs to be elucidated. miR-194 is conserved across species, including humans and dogs [19]. Dogs share the same sequence as human miR-194-5p, located in dogs on chromosome 38 [19].

*In silico* analyses demonstrate that the MAPK1 gene transcript is target of miR-194 in PBMCs from dogs with leishmaniasis [15]. MAPK1 can act on various elements of the immune response [20]. The MAPK1 gene encodes extracellular signal-regulated protein kinase 2 (ERK2), a member of the mitogen-activated protein (MAP) kinase family. ERK2 acts in the differentiation of helper T cells and regulatory T cells, inducing a decrease in T-bet and increasing the expression of GATA3 and Foxp3 [21]. In VL caused by *Leishmania donovani*, parasite downregulation of MAPK1 is associated with resistance to treatment with Antimonials [22,23]. In ERK1/2-deficient murine macrophages infected *in vitro* with *Leishmania amazonensis*, a decrease in phagocytosis and TNF-α production and an increase in IL-10 production were observed [24]. These findings demonstrate that ERK MAPK1 pathway can regulate T-bet, GATA3, FoxP3 expression and TNF-α and IL-10 production which participate immune response against *Leishmania infantum* infection, however, the regulation of these mechanisms by miR-194 in CanL has not yet been demonstrated.

Another transcript predicted as a target of miR-194 in CanL is cytokine signaling suppressor protein 2 (SOCS2) [15]. This protein is a member of the SOCS family whose primary activity is the attenuation of cytokine-triggered signal transduction [25]. No changes in parasite load and cytokine production were observed in lymph node cells from SOCS2 knockout mice infected with *L*. *major* [26]. There are no studies on the role of SOCS2 in VL; however, the absence of SOCS2 in mice stimulated the differentiation of TCD4 cells to the Th2 profile in the spleen, with increased production of IL-4, IL-10, and GATA3 [27]. Furthermore, the absence of SOCS2 also decreases the expression of the Th1 cytokine IFN-γ in the spleen and heart of mice infected with *Trypanosoma cruzi*. Together, these data suggest that SOCS2 inhibition promotes an increase in IL-4 and IL-10 cytokines and the differentiation of T helper cells to Th2, a response profile associated with the progression of CanL, however, whether miR -194 regulate Th2 in sick dogs remains to be verified.

TRAF6 transcript is also a predicted target of miR-194 in CanL [15]. The TNF receptor-associated factor 6 protein (TRAF6), participates in signal transduction after stimulation of TNF family receptors and in the TLR, OPGL, and CD40 signaling pathway [28,29], resulting in NF-κB activation and pro-inflammatory cytokine production [30,31]. TRAF6 participates in NF-κB activation in murine macrophages infected by *Leishmania donovani* [32,33], and NF-κB activation promotes the expression of TNF-α and iNOs in infected cells [32]. TRAF6 is a target of miR-194 [34,35], and its inhibition positively regulated the expression of TRAF6 and the production of cytokines IL-1, IL-6, and TNF-α in disk pulposus cells of rats [35] and TNF-α and TGF-β in a human monocytic lineage [34]. These studies suggest an important role for TRAF6 in regulating the pro-inflammatory response and activation of leishmanicidal mechanisms in infected cells, however, whether the same mechanisms are regulated by TRAF6 in CanL, as well as the regulation of TRAF6 by miR-194, still needs to be determined elucidated in the disease in dogs.

In this study, we determined whether miR-194 regulates parasite load. We measured the expression of transcription factors T-bet, GATA3, and FoxP3 and studied the leishmanicidal elements iNOS and NO in PBMCs from dogs with lesihamiasis. We measured production of cytokines IL-1β, IL-4, IL-6, IL-10, TNFα, IFN-γ, and TGFβ in culture supernatants. We also analyzed the expression of TRAF6 in these cells. We demonstrated that an increase in miR-194 activity increases parasite load, and its inhibition increases IL-1β and NO in PBMCs from dogs with leishmaniasis. We also demonstrated that increase in NO levels following reduction of miR-194 activity depended on IL-1β in PBMCs from diseased dogs.

## Methods

### Ethics statement

The Animal Experimental Research Ethics Committee approved the study, and we received the approval of the Animal Use Ethics Committee of UNESP–Universidade Estadual Paulista "Júlio de Mesquita Filho” (process number 00624–2018).

### Canine screening and sample collection

To compose the infected group, we selected 28 adult dogs, of various breeds, male and female with natural leishmaniasis infections. The infected dogs came from the Zoonosis Control Center of Araçatuba/SP Brazil and presented with positive serological tests for anti-leishmania antibodies, according to indirect enzyme-linked immunosorbent assay (ELISA) [36] and immunochromatographic (rapid test DPP /Bio-Manguinhos, BR), and for *Leishmania* spp. DNA according to qPCR [37]. All dogs were symptomatic and exhibited at least three typical clinical signs of the disease in dogs, including onychogryphosis, lymphadenopathy, hepatomegaly and splenomegaly, cachexia, alopecia, periocular lesion, and skin lesions.

We selected 5 healthy dogs of various breeds, male and female for the control group. The dogs came from private owners after providing a consent form. Healthy dogs did not show clinical, hematologic, or biochemical alterations, and the serological and molecular tests described above were negative for leishmaniasis.

Blood samples were collected by jugular vein puncture, and 8 mL were placed in plastic tubes containing K_2_EDTA (Becton-Dickson, USA), intended for obtaining mononuclear cells and carrying out the hemogram. Another 4 mL was placed in plastic tubes without anticoagulant for serology and biochemical measurements.

### PBMC isolation

PBMCs from the dogs were isolated using a gradient of Histopaque 1077 (Sigma, USA) following the manufacturer’s instructions. Red cells were lysed using a lysis buffer containing 7.46 g/L of ammonium chloride and washed three times in phosphate-buffered saline (PBS) pH 7.2. Cells were resuspended in 1 mL of RPMI 1640 supplemented with 10% fetal bovine serum, penicillin-G 100 IU/mL, streptomycin 100 μg/mL, and L-glutamine 2 mmol/L (Sigma, USA).

### DNA extraction and determination of Leishmania species in dogs with leishmaniasis

The extraction of 1x10^6^ total DNA from PBMCs from dogs with leishmaniasis was performed using a commercial DNAeasy kit (Qiagen, Valencia, California, 91355, USA) following the manufacturer’s instructions. The final DNA elution was 30 μL. The Leishmania spp. was determined using PCR-RFLP [38], comparing the sample restriction profile with the profile obtained from *Leishmania infantum* (IOC/L0575-MHOM/BR/ 2002/LPC-RPV). We used a positive control and water as a negative control.

### PBMC miRNA extraction and miR-194 expression

Total RNA from PBMC miRNAs was extracted using the mirVana miRNA Isolation kit (Invitrogen, CA, USA), following the manufacturer’s instructions. Isolated RNAs were then analyzed on a NanoDrop ND-1000 spectrophotometer (NanoDrop, Thermo Fisher, MA, USA) for purity evaluation (260/280) and quantification.

For miR-194 expression, we used a methodology described elsewhere [15]. Briefly, cDNA was synthesized using a miScript RT II kit (Qiagen, MD, USA), as recommended by the manufacturer. Next, RT-qPCR was performed using a commercially available primer specific for *Canis familiaris* miR-194 (Qiagen, MD, USA). Amplification conditions consisted of an activation step of 95°C for 15 min, followed by 40 cycles of 94°C for 15 s, 55°C for 30 s, and 70°C for 30 s for denaturation, annealing, and extension, respectively. We also generated a ten-fold standard curve using a cDNA pool serial dilution to evaluate reaction efficiency.

Because SNORD96A and miR-194 presented similar reaction efficiencies, miR-194 relative expression values were obtained using the 2^-ΔΔCt^ method [39]. All samples were tested in duplicate.

### Transfection with miR-194 mimic and inhibitor in PBMCs

miR-194 activity was altered in PBMCs of dogs using transfection of chemically-modified double-stranded RNA that mimics or inhibts microRNA with HiPerfect Transfection Reagent (QIAGEN, USA). Following the manufacturer’s recommendations, PBMCs at 1.6 x10^5^ were transfected with All Stars Negative control siRNA (5 nM), miR-194 control transfection (Scrambled), miR-194 mimic (5 nM) (Mimic), or miR-194 inhibitor (50 nM) (Inhibitor) (miScript miRNA Mimic and Inhibitor Qiagen, USA). For the evaluation of the transfection rate, siRNA AllStars HS Cell Death Control (50nM) (Qiagen, USA) was used, and cell death was analyzed using light microscopy after staining with trypan blue according to the manufacturer’s recommendations. Transfected cells were placed in culture in 24-well plates with complete RPMI medium at 37°C in a 5% CO_2_ incubator. After 48 hours, the cells and supernatants were collected to evaluate transfection rate, cell viability, and evaluation of targets of miR-194. Mean transfection rates were 20% in the control group and 22% in the infected group.

### DNA extraction and Leishmania infantum parasitic load quantification

PBMC DNA was extracted using a phenol-chloroform protocol [40]. Extracted DNA was analyzed on the NanoDrop ND-1000 spectrophotometer for purity evaluation (260/280) and quantification.

For *Leishmania infantum* parasitic load quantification, qPCR was used, employing primers that amplify the intergenic spacer internal transcript (ITS1) segment of the parasite rRNA gene at a concentration of 10mM (5’TCCAGCACATTTTGCGA GTA3’ and 5’CCACACAGGTTTCTTCTTTATTTGG3’) [41]. qPCR reaction was standardized with 30 ng purified genomic DNA, 12.5 μL SYBR Green JumpStart Taq ReadyMix (Sigma-Aldrich, St. Louis, MO, USA), 5 pmol of each primer, and 9.5 μL of ultrapure H_2_O in a final reaction volume of 25 μL. We also generated a ten-fold standard curve using extracted DNA from *Leishmania infantum* promastigotes (MHOM/BR00/MER02) for parasite quantification. Reactions were performed using a Mastercycler RealPlex2 system (Eppendorf, CT, USA) under the following conditions: Initial heating of 94°C for 2 min, followed by 40 cycles of denaturation (94°C for 15 s), annealing, and extension (58°C for 1 min). After these steps, a dissociation curve of the amplified fragment was determined (95°C for 15 s, then 60°C to 95° C at 15 s/°C). Parasitic DNA load was determined in each sample by comparing each sample to the standard curve.

### Quantification of parasite load, NO, iNOS, T-bet, GATA3, and FoxP3 using flow cytometry

To quantify the parasite load, the method described by Di Giorgio et al. [42] was used with modifications. After 48 hours of culture at 37° C and 5% CO_2_, PBMCs were harvested and incubated for 60 minutes at room temperature in 1x PBS containing 1% paraformaldehyde (Sigma). Cells were centrifuged at 400 *g* for 5 minutes. Permeabilization of cell membranes and parasitophorous vacuoles was performed by resuspending the cells in ethanol (Sigma) for 60 minutes at –20° C. Cells were washed with 2% bovine serum albumin (BSA, Sigma) in 1x PBS and incubated for 60 minutes at 4°C with anti-LPG *Leishmania* monoclonal antibody (ABD, Serotec) diluted 1/250. After three successive washes with PBS/BSA, amastigotes were stained with 1 μM of PE-conjugated anti-IgG2a (Sigma) and FITC-conjugated anti-human CD14 monoclonal antibody (Bio-Rad) for 60 minutes at 4° C. Cells were labeled for monocytes (CD14+ cells) and positivity for gp63.

To determine intracellular NO levels, PBMCs were centrifuged at 400 *g* for 5 minutes, resuspended in a minimum volume of PBS, and stained with DAF-2DA (2M) (Invitrogen-Leiden Molecular Probes, Netherlands) for 60 minutes at 37° C in the presence of 5% CO_2_. Samples without markings were used as a negative control to delimit the negative populations of the analyzed samples.

For analysis of iNOS production, PBMCs were fixed with 500 μl of fixation buffer (Invitrogen) and incubated for 10 min at room temperature. Cells were centrifuged at 400 *g* for 5 minutes and washed twice with permeabilization buffer (Invitrogen). Then, PBMCs were resuspended with 50 μl of permeabilization buffer (Invitrogen) and incubated with anti-human iNOS antibodies conjugated to PE and its respective isotype control (BIORBYT) for 60 minutes at 4° C. Cells were washed with PBS/BSA and stored at 4°C in the dark until analysis.

To determine the expression of T-bet, GATA3, and FoxP3 transcription factors, T CD3+ lymphocytes were incubated. PBMCs were suspended in PBS containing 1% BSA, 0.1% azide, and 20% FBS to block Fc receptors and incubated for 30 minutes at room temperature. Cells were centrifuged at 400 *g* for 5 minutes and incubated with FITC-conjugated Anti-Dog CD3 monoclonal antibodies and their respective isotype controls (Bio-Rad, USA). PBMCs were then fixed with 500 μl of fixation buffer (Invitrogen) and incubated for 45 minutes at room temperature. Cells were centrifuged at 400 *g* for 5 minutes and washed twice with permeabilization buffer (Invitrogen). Then, cells were resuspended with 50 μl of permeabilization buffer (Invitrogen) and incubated with anti-human T-bet (BioLegend, USA), anti-human GATA3 (BioLegend, USA), and anti-human Foxp3 (eBioscience) antibodies conjugated to PE and with their respective isotype control during 90 minutes at 4° C. Cells were washed with PBS/BSA and stored at 4° C in the dark until analysis.

For all targets, the acquisition of 10.000 events was performed on an Accuri C5 flow cytometer (BD Biosciences, USA) and analyzed using BD Accuri C6 software, version 1.0.264.21 (BD Biosciences, CA, USA).

### Quantification of cytokines IL-1β, IL-4, IL-6, IL-10, TNF-α, IFN-y, and TGF-β by capture ELISA

Detection of cytokines in the PBMC culture supernatants was performed using the DuoSet ELISA Development Systems kits (R&D Systems, Minneapolis, MN, USA), according to the manufacturer’s instructions.

### Relative gene expression of TRAF6

After isolation of PBMCs, total RNA was extracted from PBMCs using a commercial RNeasy Mini Kit (Qiagen, Valencia, California, 74104, USA) according to the manufacturer’s recommendations. Then, RNA was eluted in nuclease-free water, and the concentration of the extracted RNA (ng/μL) and the degree of purity (A260 nm/A280 nm coefficient) were evaluated using a spectrophotometer (NanoDrop Technologies ND 1000 UV/VIS, USA). Cells were frozen at –80°C to perform reverse transcription subsequently. cDNA production was performed using a commercial QuantiTect Reverse Transcription kit (Qiagen, Valencia, California, 205311, USA) with 100 ng of RNA and oligo (dT) primer in a final volume of 20 μL. cDNA was frozen at –20°C until analysis.

TRAF6 gene expression was determined by RT-qPCR using a Mastercycler-Ep Realplex 4-S thermal cycler (Eppendorf North America, Westbury, NY, USA). The RT-qPCR reactions were standardized with 1ul of cDNA, 4 μl of HOT FIREPoL EvaGreen qPCR MIX Plus (no ROX) 5X (SOLIS BIODYNE), 100 nM of each oligonucleotide primer, and 13.2μl of ultrapure water in a volume of final reaction of 20 μl. Specific primers for TRAF6 were designed using the Primer3Plus1 software (UNTERGASSER et al., 2012), selecting the fragment 5’TCTGCAAAGCTTGCATCA TC3’ and 5’AAGGTACGTTGGCACTG GAG3’. Amplification conditions were an initial incubation of 2 min at 50°C and 2 min at 95°C, followed by 40 cycles at 95°C for 15 s, 60°C for 1 min, and 60°C for 1 min. At the end of amplification, a dissociation curve of the amplified fragment was determined under the following conditions: 95°C for 15 s, 60°C for 15 s, followed by 20 minutes until reaching 95°C for 15 s. Nuclease-free water (Sigma-Aldrich Co, St. Louis, USA) was used as a negative control, and samples were evaluated in duplicate. Reaction efficiency values, coefficients of determination, and angular coefficients (slopes) were obtained from the amplification of seven serial dilutions of a cDNA pool. To analyze the results, the relative gene expression was performed with efficiency correction as previously described [43], using the geometric mean of reference genes beta-actin (5’CCAGCAAGGATGAAGATCAAG3’ and 5’TCTGCTGGAAGGTGGACA G3’) and HPRT-1 (5’CACTGGGAAAACAATGCAGA3’ and 5’ACAAAGTCAGGTT TATAGCCAACA3’) [44].

### Functional assay with IL-1β blockade after transfection with miR-194 inhibitor in PBMCs from dogs with leishmaniasis

For the functional assay with IL-1β neutralization, PBMCs from dogs with leishmaniasis were transfected with miR-194 Inhibitor as described in section 2.4.5, and treated with canine IL-1β/IL-1F2 beta (R&D System) at 400 ng/mL with its respective isotype control (Sheep IgG Isotype Control-Invitrogen/USA) according to the manufacturer’s recommendations. Cells were cultured in 24-well plates with complete RPMI medium at 37°C and 5% CO_2_. After 48 hours, cells were stained with DAF-2DA (2M) (Invitrogen-Leiden Molecular Probes, The Netherlands) for 60 minutes at 37°C in 5% CO_2_ for NO detection. IL-1β was evaluated in PBMCs culture supernatants by capturing ELISA to assess blocking efficiency (S1 Fig).

### Statistical analysis

Statistical analysis was performed using GraphPad Prism v6 software (GraphPad Software, Inc., La Jolla, CA, USA). All statistical variables were tested for normality using the Shapiro-Wilk test. To analyze the correlation between the expression of miR-194 and the relative gene expression of TRAF6, the PEARSON correlation test was used. To compare the values corresponding to the parasite load and production of iNOS, NO, T-bet, GATA3, FoxP3, IL-1β, IL-4, IL-6, IL-10, TNF-α, IFN-y, TGF-β, and TRAF6 expression between treatments within groups, Friedman’s test with Dunn’s post-test was used. Mann-Whitney test was used to compare the results between the control and infected groups. Values were considered significant when p < 0.05.

## Results

### All dogs with leishmaniasis were infected with *Leishmania infantum*

To determine the species of *Leishmania* present in the infected dogs, Restriction fragment polymorphism (RFLP)-PCR was performed. All sick dogs (Infected group) were infected with *Leishmania infantum* (S2 Fig).

### Clinical and laboratory findings

Dogs with leishmaniasis (Infected group) showed at least three characteristic clinical signs of the disease, the most frequent clinical sign being skin lesions (86% [24/28]), followed by lymphadenopathy (75% [21/28]), onychogryphosis (68% [19/28]), cachexia (50% [14/28]), seborrhea (36% [10/28]), alopecia and periocular lesions (32% [9/28]), and hepatosplenomegaly (28% [8/28]). Healthy dogs (Control group) showed no clinical signs (S1 Table). Dogs in the infected group tested positive for the anti-*Leishmania* antibodies by indirect ELISA [36] and the rapid DPP test. They tested positive for *Leishmania* spp. DNA by qPCR (S1 Table). Healthy dogs (Control group) were negative for all serological and molecular diagnostic tests for leishmaniasis (S1 Table).

Dogs with leishmaniasis showed a significantly lower red blood cell count, hemoglobin, hematocrit, and serum albumin concentration and significantly higher serum globulin concentrations than healthy dogs (control group) (S2–S4 Tables). Based on clinical and laboratory findings, sick dogs showed moderate manifestations of leishmaniasis and were classified in stage II of the disease, as proposed by Solano-Gallego et al. [45]. Healthy dogs (control group) showed no alterations in the blood count (S1 and S2 Tables) or biochemical parameters (S4 Table).

### miR-194 is increased in PBMCs from dogs with leishmaniasis

**Fig 1:**
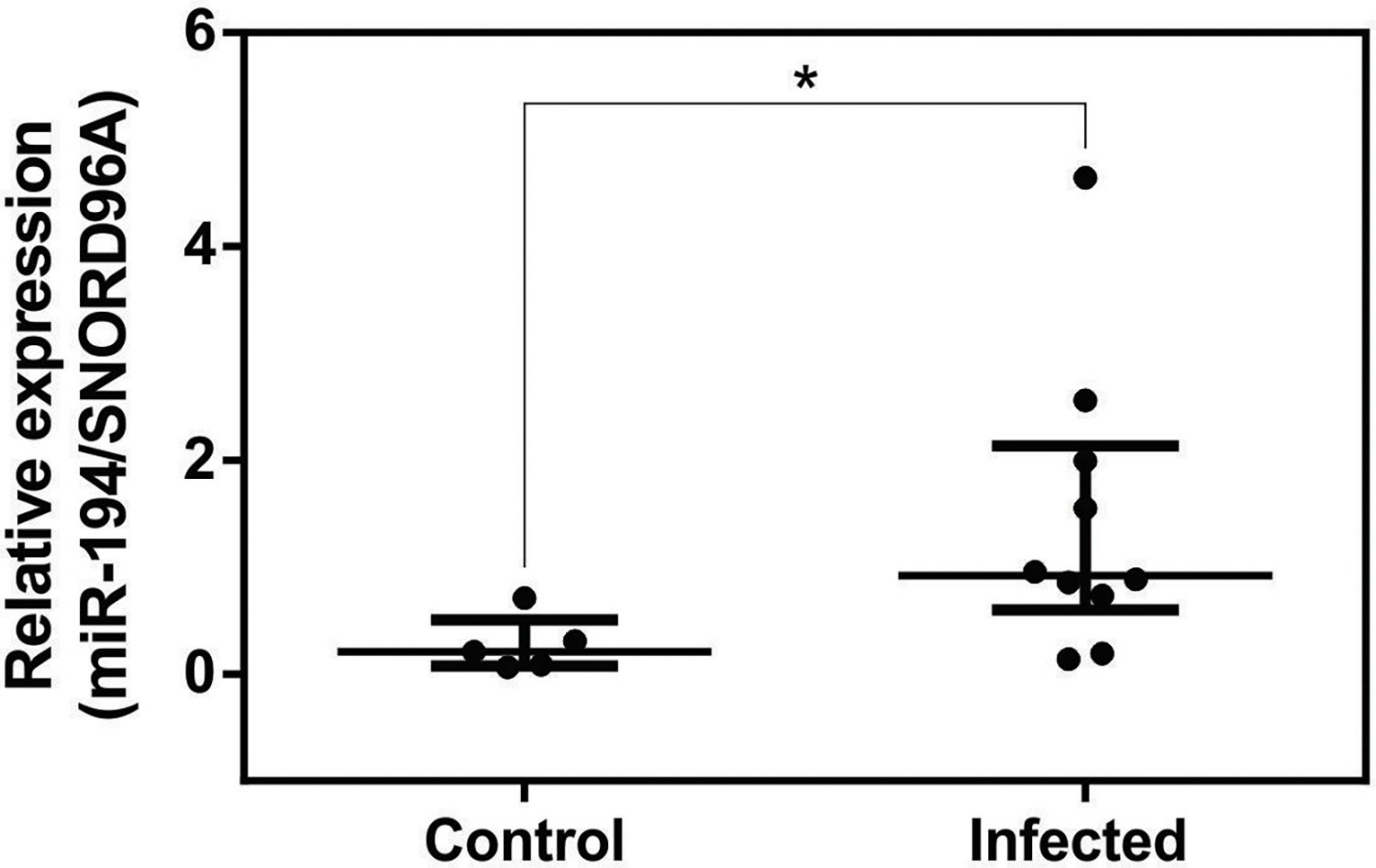
miR-194 expression in PBMCs from healthy dogs and those with leishmaniasis. Expression of miR-194 in PBMCs from healthy dogs (Control Group N = 5) and dogs with leishmaniasis (Infected Group N = 10) was quantified using qPCR. Data are expressed as the median and interquartile range (25 and 75). Symbols represent individual data for each animal. Asterisks indicate significant differences (Mann-Whitney test, p < 0.05).

miR-194 expression was elevated in the PBMCs of dogs with leishmaniasis [15]. To validate the increase in miR-194, we measured the expression of miR194. Expression of miR-194 was significantly higher in the PBMCs of dogs in the infected group than in the Control group (Fig 1).

### Mimic miR-194 increased parasite load in PBMCs from dogs with leishmaniasis

**Fig 2:**
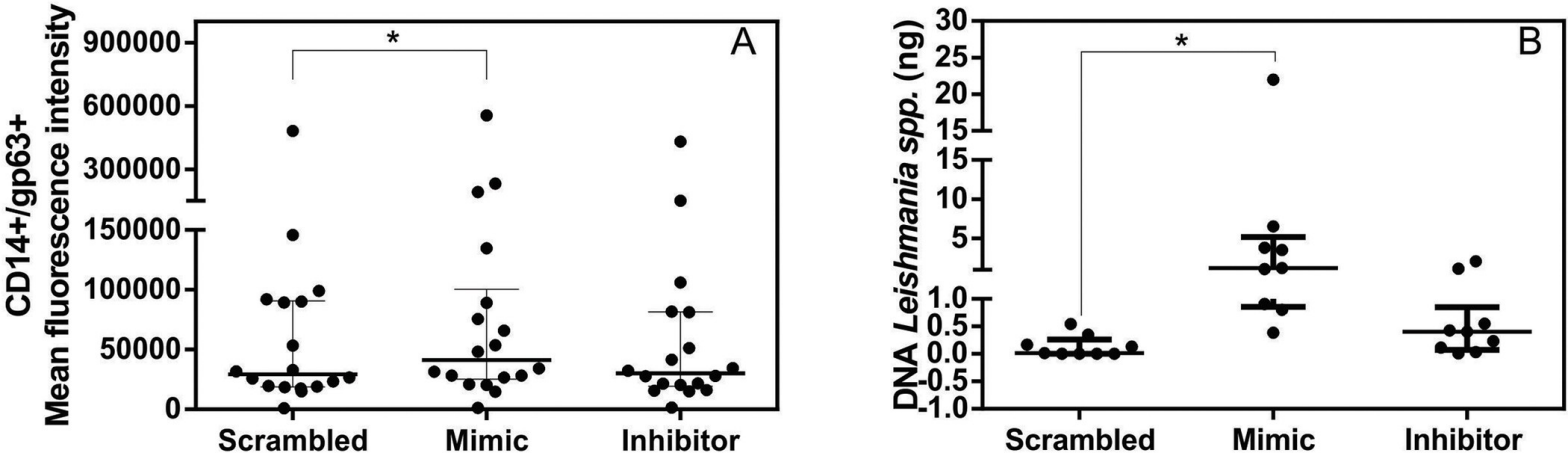
Quantifying parasite load in PBMCs from dogs with leishmaniasis after transfection with Mimic and miR-194 Inhibitor. Parasite load was quantified using flow cytometry (N 18) (A) and qPCR (N = 9) (B). Data are expressed as the median and interquartile range (25 and 75). Symbols represent individual data for each animal. Asterisks indicate significant differences (Friedman’s test followed by Dunn’s test, p < 0.05).

Progression and worsening of CanL are associated with parasite proliferation [46]. miR-194 was positively correlated with increased parasite load in the PBMCs of dogs with leishmaniasis [15]. To determine the role of miR-194 in regulating parasite burden, PBMC from diseased dogs were transfected with miR-194 mimic and miR-194 inhibitor, and after 48 hours of culture at 37°C and 5% CO_2_, parasite burden was determined. Parasite load increased after transfection with miR-194 mimic according to flow cytometry (Fig 2A) and quantification of *Leishmania* spp. DNA using qPCR (Fig 2B). Flow cytometry gating strategy data for parasite load is shown in S3 Fig

### Inhibitor miR-194 increased NO levels in PBMCs from dogs with leishmaniasis

**Fig 3:**
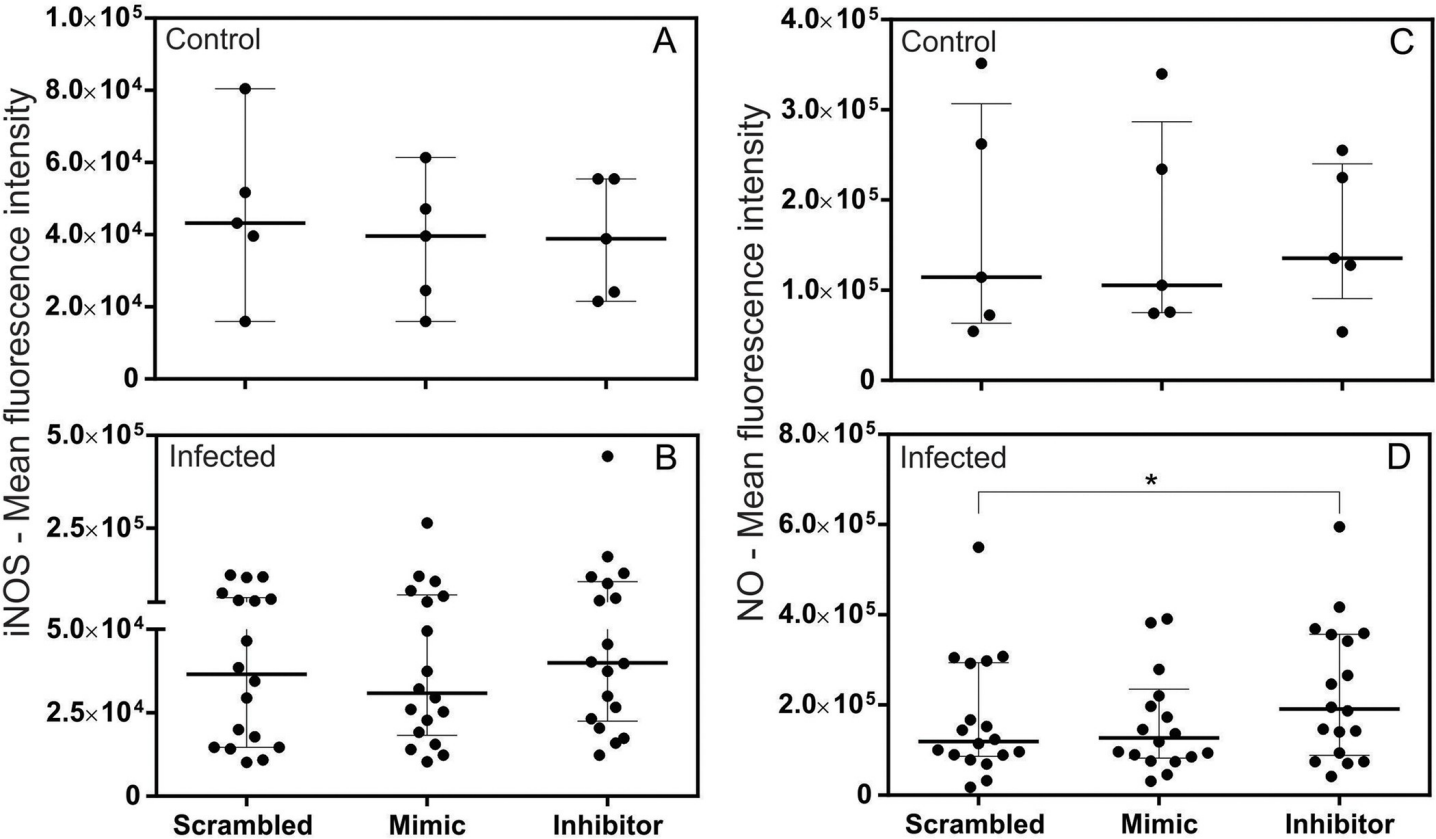
iNOS and NO production in PBMCs from healthy dogs and those with leishmaniasis after transfection with Mimic and Inhibitor of miR-194. The evaluation of iNOS and NO production was performed on PBMCs from healthy dogs (Control group, N = 5) (A and C) and dogs with leishmaniasis (Infected group, N = 18) (B and D). Data are expressed as the median and interquartile range (25 and 75). Symbols represent individual data for each animal. Asterisks indicate significant differences (Friedman’s test followed by Dunn’s test, p < 0.05).

In CanL, parasite control depends on the activation of leishmanicidal mechanisms such as iNOS activation [47,48] and NO production in infected cells [49–51]. To determine whether miR-194 regulates iNOS and NO production, PBMCs from healthy dogs and those with leishmaniasis were transfected with Mimic and Inhibitor of miR-194. After 48 hours of culture at 37° C and 5% CO_2_, spontaneous production of iNOS and NO was measured using flow cytometry. There were no significant differences in basal iNOS and NO production between PBMCs from healthy dogs and those with leishmaniasis (S4 Fig). The same result was observed for iNOS and NO production in PBMCs from healthy dogs after increasing and decreasing miR-194 activity (Fig 3A and 3C). Interestingly, there was a tendency to increase iNOS production, followed by a significant increase in NO production after transfection with a miR-194 inhibitor in PBMCs from dogs with leishmaniasis (Fig 3B and 3D). Flow cytometry gating strategy data for iNOS and NO production is shown in S5 Fig).

### miR-194 did not regulate the expression of transcription factors T-bet, GATA3, or FoxP3 in PBMCs from dogs

**Fig 4:**
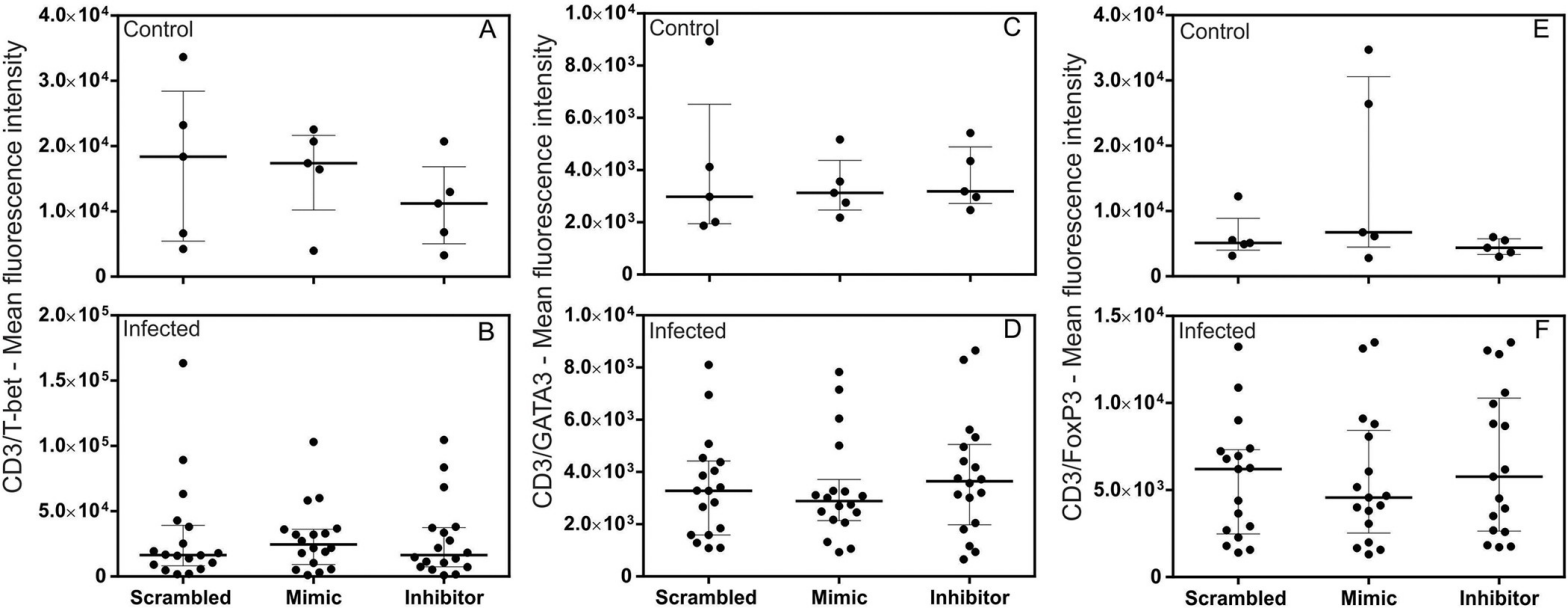
Quantification of T-bet, GATA3, and FoxP3 transcription factors in lymphocytes from healthy dogs and those with leishmaniasis after transfection with miR-194 Mimic and Inhibitor. Quantification of T-bet, GATA3, and FoxP3 transcription factors was performed on PBMCs from healthy dogs (Control group, N = 5) (A, C, and E) and dogs with leishmaniasis (Infected group, N = 18) (B, D, and F). Data are expressed as the median and interquartile range (25 and 75). Symbols represent individual data for each animal. Asterisks indicate significant differences (Friedman’s test followed by Dunn’s test, p < 0.05).

Progression of leishmaniasis in dogs is associated with an exacerbated humoral immune response (Th2) or the development of an immunosuppressive state at the expense of an effective cellular immune response (Th1) [1,52,53]. Treg cells have been associated with a protective role during infection in dogs [54–56]. Generation of subsets of Th1, Th2, and Treg cells involves the expression of transcription factors T-bet [57], GATA3 [58], and FoxP3 [59], respectively. To determine whether there is a regulatory role for miR-194 in the differentiation of TCD4+ cells subsets, PBMCs from healthy dogs and those with leishmaniasis were transfected with miR-194 Mimic and Inhibitor and cultured for 48 hours at 37° C and 5% CO_2_. There was no significant difference in the basal expression of transcription factors between healthy dogs and those with leishmaniasis (S6 Fig). The same result was observed with the increase or decrease of miR-194 activity in T-bet expression (Fig 4A and 4B), GATA3 (Fig 4C and 4D), and FoxP3 (Fig 4E and 4F). Flow cytometry gating strategy data for T-bet, GATA3 and FoxP3 expression is shown in S7 Fig.

### miR-194 Inhibitor increased production of IL-1β in the supernatants of PBMC culture from dogs with leishmaniasis but did not alter the production of IL-6, IL-4, TNF-α, IFN-y, TGF-β, or IL -10

Pro-inflammatory cytokines such as IL-1β, IL-6, TNF-α, and IFN-y have been associated with resistance in leishmaniasis [1,60]. In contrast, immunoregulatory cytokines such as TGF-β and IL-10 and anti-inflammatory such as IL-4 are associated with disease susceptibility [52,61].

Test for a possible regulatory role of miR-194 in the production of cytokines IL-1β, IL-6, IL-4, TNF-α, IFN-y, TGF-β, and IL-10, PBMCs from healthy dogs and those with leishmaniasis were transfected with miR-194 Mimic and Inhibitor. After 48 hours of culture at 37° C and 5% CO_2_, supernatants were collected, and the cytokines were measured using capture ELISA.

IL-4 and TGF-β were increased in the PBMCs supernatants from dogs with leishmaniasis, and the other cytokines did not show significant differences between the groups (S8 Fig).

**Fig 5:**
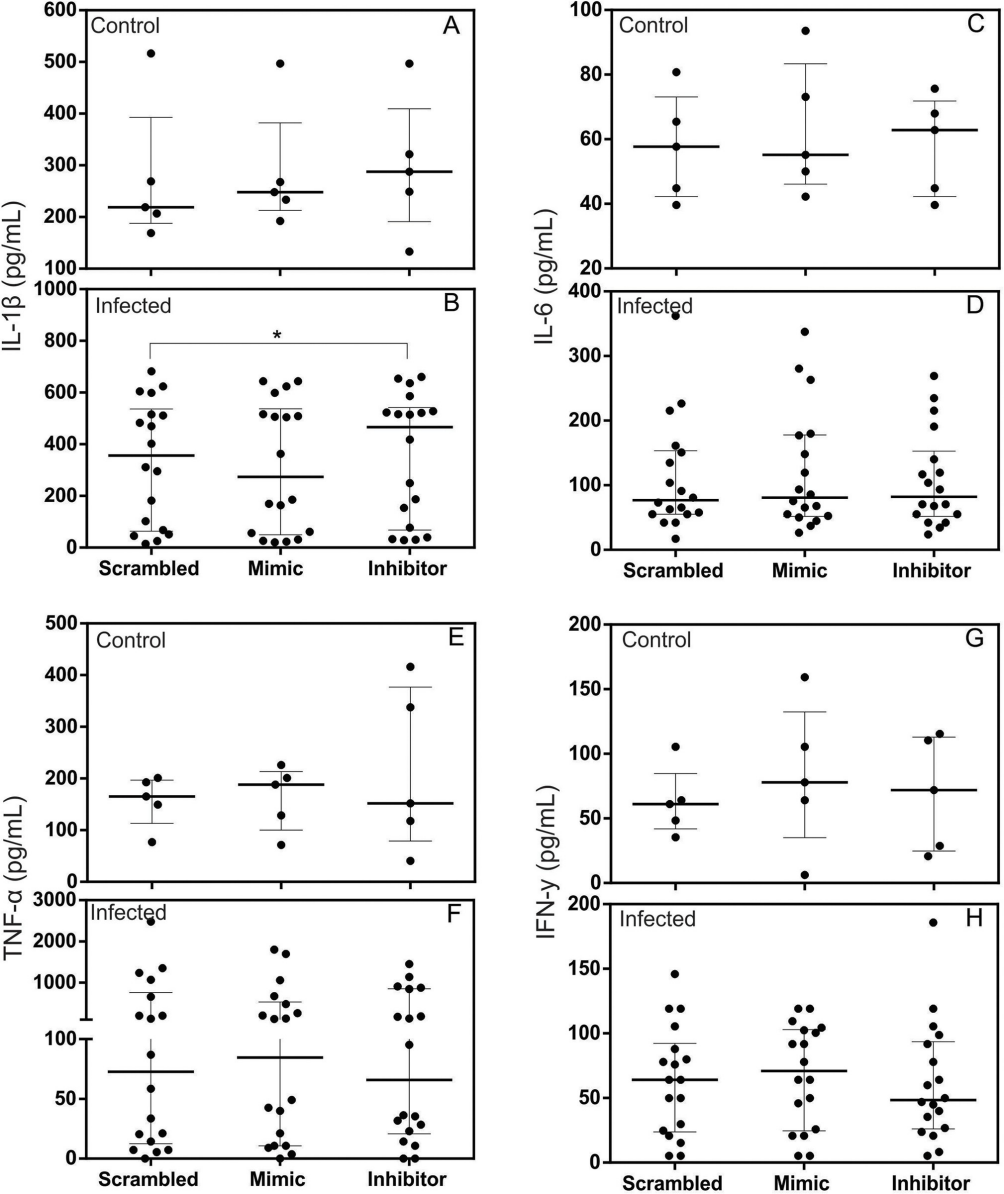
Quantification of pro-inflammatory cytokines in the supernatants of cultured PBMCs from healthy dogs and those with leishmaniasis after transfection with miR-194 Mimic and Inhibitor. Production of pro-inflammatory cytokines IL-1-β, IL-6, TNFα and IFN-y was performed on the PBMC culture supernatants of healthy dogs (Control group, N = 5) (A, C, E and G) and dogs with leishmaniasis (Infected group, N = 18) (B, D, F and H). Data are expressed as the median and interquartile range (25 and 75). Symbols represent individual data from each animal. Asterisks indicate significant differences (Friedman’s test followed by Dunn’s test, p < 0.05).

**Fig 6:**
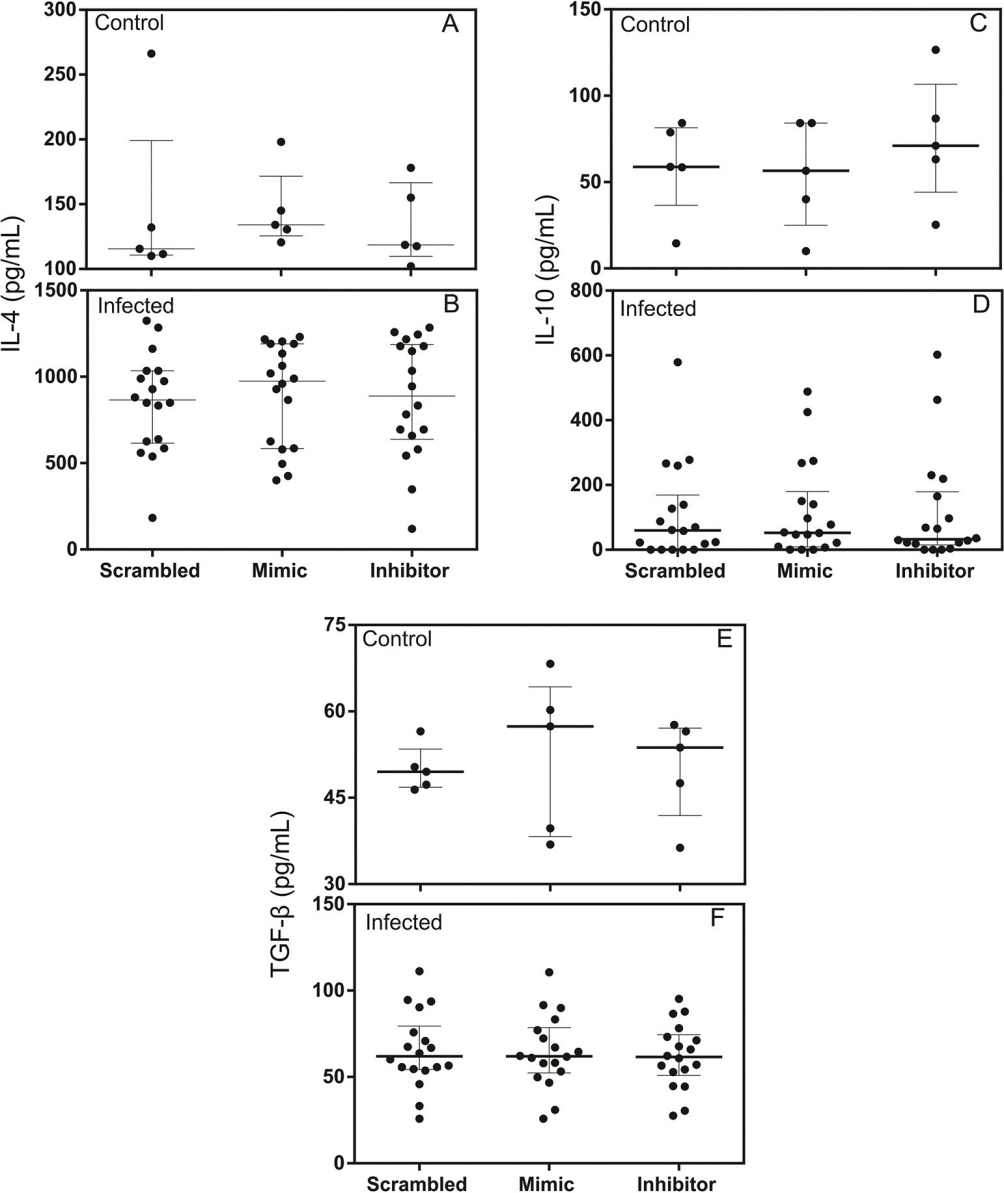
Quantification of anti-inflammatory and immunoregulatory cytokines in the supernatants of cultured PBMCs from healthy dogs and dogs with leishmaniasis after transfection with miR-194 Mimic and Inhibitor. Production of anti-inflammatory cytokine IL-4 and immunoregulatory cytokines IL-10 and TGF-β, were evaluated using capture ELISA. The evaluation was performed on the PBMC culture supernatants of healthy dogs (Control group, N = 5) (A, C, and E) and dogs with leishmaniasis (Infected group, N = 18) (B, D, and F). Data are expressed as the median and interquartile range (25 and 75). Symbols represent individual data from each animal. Asterisks indicate significant differences (Friedman’s test followed by Dunn’s test, p < 0.05).

Regarding the pro-inflammatory cytokines IL-1β, IL-6, TNF-α, and IFN-y, only IL-1β increased in PBMC culture supernatants from dogs with leishmaniasis after transfection with the miR-194 inhibitor (Fig 5B). In contrast, of healthy dogs post transfection, there was no alteration in IL-1β in the PBMC supernatants (Fig 5A). No changes were observed in the production of IL-6 (Fig 5C and 5D), TNF-α (Fig 5E and 5F), or IFN-y (Fig 5G and 5H) in the PBMC culture supernatants of healthy dogs and those with leishmaniasis after transfection with miR-194 Mimic and Inhibitor. This same result was found for the production of the anti-inflammatory cytokine IL-4 (Fig 6A and 6B) and the immunoregulatory cytokines IL-10 (Fig 6C and 6D) and TGF-β (Fig 6E and 6F).

### miR-194 expression is negatively correlated with the relative gene expression of TRAF6 in PBMC from dogs with leishmaniasis

**Fig 7:**
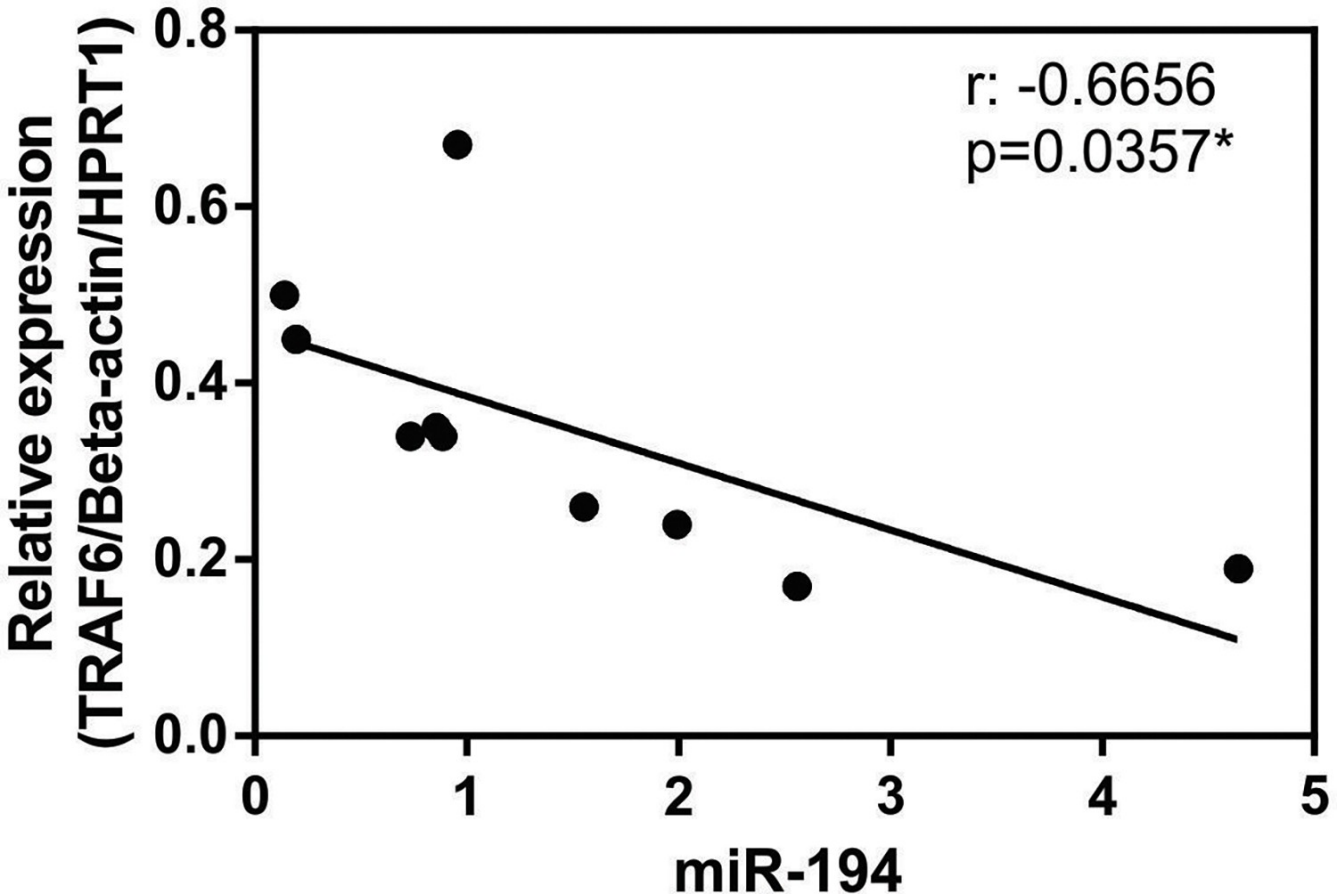
Correlation between miR-194 expression and relative gene expression of TRAF6 in PBMC from dogs with leishmaniasis. Expression of miR-194 and relative expression gene of TRAF6 in PBMCs from dogs with leishmaniasis (N = 10) was quantified using qPCR. Data represents a negative correlation between miR-194 expression and relative gene expression of TRAF6. Asterisks indicate significant correlation by Pearson correlation test.

*In silico* analyzes demonstrated that TRAF6 is a target of miR-194 in the PBMCs of dogs with leishmaniasis [15]. In rat spinal disk pulposus cells, miR-194 regulates TRAF6 translation [35]. Relative expression of TRAF6 was lower in PBMCs from dogs with leishmaniasis than those from healthy dogs (S9 Fig). To assess whether miR-194 plays a role in regulating TRAF6 expression, we analyzed the correlation between miR-194 expression and TRAF6 in PBMC from dogs with leishmaniasis. There is a significant negative correlation between miR-194 expression and TRAF6 expression in PBMCs from dogs with leishmaniasis (Fig 7A). The TargetScan 8.0 (an in-silico miRNA target prediction tool) to predict the interaction between TRAF6 and cfa-miR-194. In a conserved region, cfa-miR-194 seed region is completely linked (8-mer site type) with the position 157–164 of TRAF6 3’ UTR and have a low context score (CS) and a CS percentile of 96. This shows us that this interaction is highly predicted to happen and is efficient.

### IL-1β blockade decreases NO after miR-194 inhibition in PBMCs from dogs with leishmaniasis

**Fig 8:**
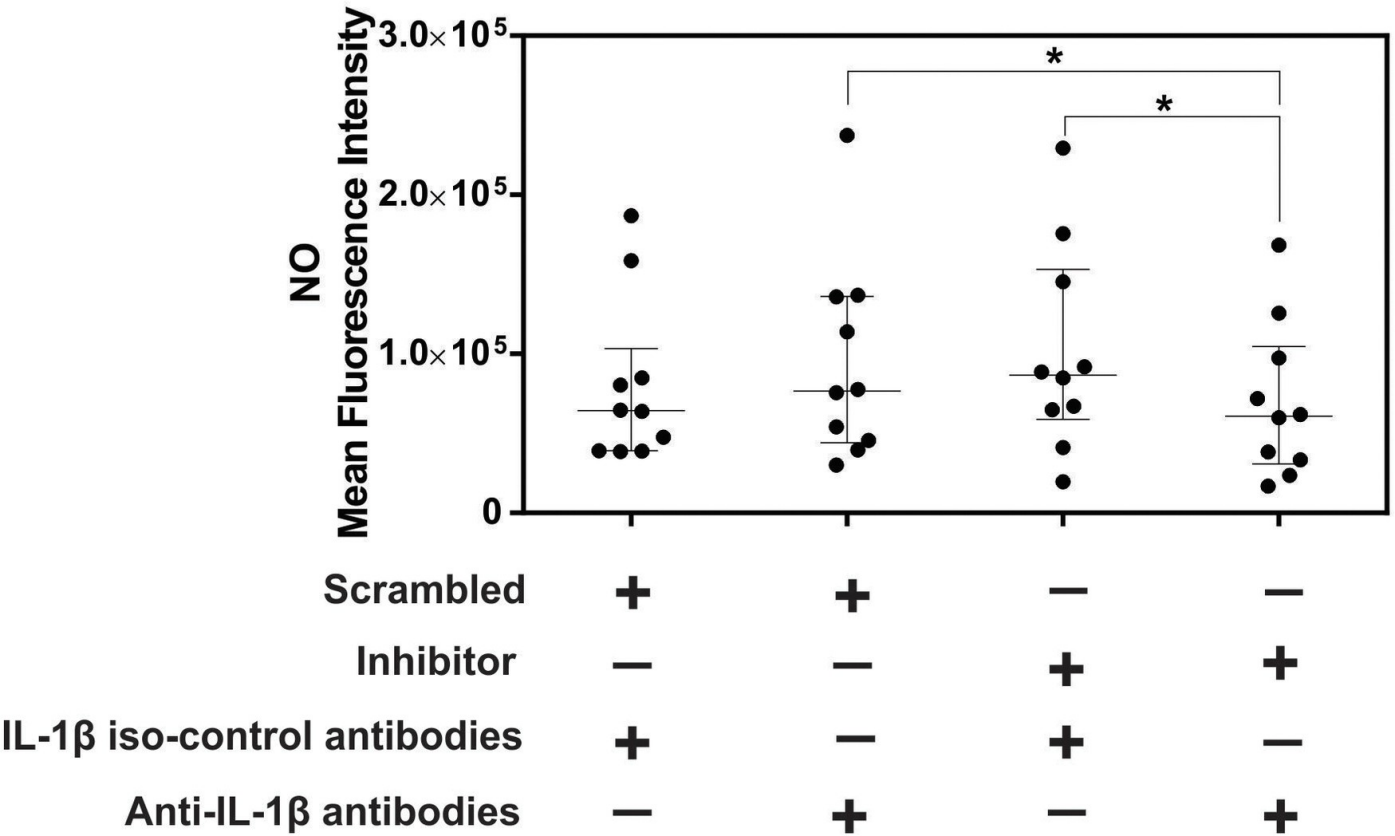
NO levels in PBMCs from dogs with leishmaniasis after transfection with miR-194 inhibitor and IL-1β blockade. The evaluation NO production after IL-1β blockade. Data are expressed as the median and interquartile range (25 and 75). Symbols represent individual data for each animal. Asterisks indicate significant differences (Friedman’s test followed by Dunn’s test, p < 0.05).

miR-194 inhibition increased IL-1β and NO production in PBMCs from dogs with leishmaniasis. Adding IL-1β to cultured macrophages from mice infected with *Leishmania amazonensis* increased nitrite production in culture supernatants [60]. To determine whether NO production depends on IL-1β in PBMCs from dogs with leishmaniasis after miR-194 inhibition, a functional assay with IL-1β blockade was performed. PBMCs from dogs with leishmaniasis were transfected with miR-194 inhibitor with IL-1β blocking antibody. After 48 hours of culture at 37° C and 5% CO_2_, NO production was evaluated using flow cytometry. Nitric oxide production was significantly decreased after transfection with miR-194 inhibitor followed by IL-1β blockade when compared to PBMC transfected with miR-194 Scramble and IL-1β blockade and also when compared with miR-194 inhibitor without IL-1β blockade (Fig 8). Flow cytometry gating strategy data for NO production is shown in S10 Fig.

## Discussion

Increased parasite load in dogs naturally infected with *Leishmania infantum* is associated with the inability to establish an effective cellular immune response (Th1) and an exacerbated stimulation of the humoral immune response (Th2). There is evidence that miR-194 positively regulates parasite load in CanL [15], possibly affecting gene expression and translation of proteins fundamental for immune system development, function, and cell differentiation. In the present study, molecular tools were used to increase and decrease miR-194 activity in PBMCs from dogs, with and without leishmaniasis, to evaluate its functional role in activating leishmanicidal mechanisms. We also measured the expression of transcription factors related to cell differentiation T helper profile Th1, Th2, and regulatory T cells, pro-inflammatory, anti-inflammatory, and immunoregulatory cytokines, and their correlation with parasitic load.

miR-194 was increased in the PBMCs of dogs with leishmaniasis. Increased activity of miR-194 using mimic increased parasite load in PBMCs of diseased dogs. In a previous study, there was an increase in miR-194 in the PBMCs of dogs with leishmaniasis, which positively correlated with parasite load [15]. There are no studies on the role of miR-194 in parasite load in CanL. In H1N1 influenza virus infection, increased miR-194 activity induced an increase in viral load *in vitro* in human lung cells, and *in vivo* in mice [62]. In both models, miR-194 downregulated the production of IFN-α and IFN-β, facilitating viral replication. These findings suggest that miR-194 participates in upregulating the replication of intracellular parasites, including CanL; however, the mechanisms by which miR-194 regulates parasite load in diseased dogs are unknown.

In CanL, high levels of iNOS and NO are associated with decreased parasitic load [63]. We observed that after miR-194 activity was decreased with an inhibitor in the PBMCs of dogs with leishmaniasis, there was an increase in iNOS followed by NO, suggesting a possible regulation of this factor by miR-194. In addition to its microbicidal effect on monocytes and macrophages, NO is produced from iNOS in other immune system cells, such as T lymphocytes [64] and dendritic cells [65]. In human and mouse T lymphocytes and dendritic cells, NO exerts several immunoregulatory effects, including a decrease in apoptosis [66] an increase in MHC II expression [67,68], and increased chemotaxis directed by CCL19 [69]. Although these mechanisms have not yet been described in CanL, it is possible that NO, in addition to its leishmanicidal effects, has immunoregulatory functions.

MAPK1 is a predicted target of miR-194 in CanL [15]. It encodes the ERK2 protein involved in various stages and components of the immune system [20]. In mice with ERK2 protein deficiency, there is increased expression of GATA3 and FoxP3 and decreased expression of T-bet in T lymphocytes of lymph nodes and the spleen [21]. In our study, miR-194 did not regulate the expression of transcription factors T-bet, GATA3, or FoxP3 in CanL, suggesting that miR-194 has no role in the MAPK1/ERK2 pathway in the expression of these transcription factors in sick dogs.

We observed higher levels of IL-4 and TGF-β in the supernatants of PBMCs from dogs with leishmaniasis than from healthy dogs, confirming the regulatory role of these cytokines in CanL. IL-4 increases in the blood of dogs infected with *Leishmania infantum* with the onset of clinical signs [70]. High levels of TGF-β have been observed in the supernatant of spleen cell cultures [71] and the lymph nodes associated with disease progression in dogs [61]. No significant change was observed in the expression of IL-4 and TGF-β or IL-10 and IL-6, TNFα, and IFN-y after increasing and decreasing miR-194 activity. These findings suggest that in CanL, miR-194 has no regulatory role in the expression of these cytokines.

By contrast, there was an increase in IL-1β following a decrease in miR-194 activity in the supernatant of PBMCs from dogs with leishmaniasis. Analysis of the sequences of the IL-1β and miR-194 in dogs in the “TargetScan” program, demonstrate that there is no complementarity between the sequences [72], suggesting an indirect regulation of IL-1β by miR-194.

Several studies showed that miR-194 decreases inflammatory response, negatively regulating the production of pro-inflammatory cytokines (e.g., IL-1β) and inhibiting the activation of the NF-κB pathway by various targets, and in various cell types [35,73,74]. miR-194 inhibits the NF-κB pathway by decreasing the expression of TRAF6 in cells of the disk pulposus of the intervertebral disk of rats [35], decreases the expression of neutrophilin 1 in human astrocytes in neurodegenerative diseases [73], and decreases the chemokine CXCR4 in murine alveolar macrophages in acute lung injury [74]. In these studies, decreasing miR-194 activity increased IL-1β production and its regulatory targets. *In silico* analyses demonstrated that TRAF6 is a target of miR-194 in PBMCs of dogs with leishmaniasis [15], and previous studies demonstrated that TRAF6 is a target of miR-194 [35,74,75]. Thus, it is possible that miR-194 indirectly regulates IL-1-β production via TRAF6 in CanL, since we observed a negative correlation between miR-194 expression and TRAF6 expression in PBMC from sick dogs.

We observed that the decrease in miR-194 activity in PBMCs from dogs with leishmaniasis increased NO and IL-1β in the culture supernatants. IL-1β regulates NO production in cells infected by intracellular protozoa [60,76]. To clarify the modulatory role of IL-1β in NO production, IL-1β blockade was performed after miR-194 activity decreased in PBMCs from dogs with leishmaniasis. IL-1β blockade significantly decreased NO production, suggesting a regulatory role for IL-1β in NO production. *Leishmania* induces NLRP3 inflammasome activation in macrophages *in vitro* and in mice *in vivo*. The production of IL-1β and the IL1-β-IL-1R and MyD88 signaling pathways increase NO production [60]. Furthermore, miR-194 can downregulate inflammasome activation in inflammatory cells by significantly decreasing IL-1β production [75]. In the present study, we did not analyze inflammasome activation following increases and decreases in miR-194 activity. The mechanism by which miR-194 regulates IL-1β in CanL remains unknown.

We conclude that miR-194 is upregulated in PBMCs from dogs naturally infected with *Leishmania infantum* and increases parasite load, possibly decreasing IL-1β-dependent NO production. These findings contribute to the understanding of the molecular mechanisms by which the parasite evades the host’s immune response, in addition to identifying targets for new therapies.

## Supporting information

S1 Fig Quantifying IL-1-β in the PBMC supernatant cultures of dogs with leishmaniasis after transfection with miR-194 Inhibitor and IL-1-β blockade. Data are expressed as the median and interquartile range (25 and 75). Symbols represent individual data for each animal. Asterisks indicate significant differences (Friedman’s test followed by Dunn’s test, p < 0.05).
(TIF)

S2 Fig RFLP analysis of ITS1-PCR fragments amplified from DNA samples of standard isolates using the Hae III enzyme. M: molecular marker (100 bp); Li: *Leishmania infantum*. Samples 1 to 28 showed an identical sampling profile to *L*. *infantum*. RFLPs were identified on 3% agarose gels stained with gel red and indicated by an arrow.
(TIF)

S3 Fig Histogram representative of the flow cytometric analysis of the labeling of parasite load. Gate M1 were used to detect CD14+ cells (A) and Gate M2 were used to detect GP63 positive CD14+ cells (B)
(TIF)

S4 Fig Basal levels of iNOS and NO in PBMCs from healthy dogs and dogs with leishmaniasis. The evaluation of NO production was performed on PBMCs from healthy dogs (Control group, N = 5) (A) and dogs with leishmaniasis (Infected group, N = 18) (B). Data are expressed as the median and interquartile range (25 and 75). Symbols represent individual data for each animal. Asterisks indicate significant differences (Mann-Whitney test, p < 0.05).
(TIF)

S5 Fig Histogram representative of the flow cytometric analysis of the labeling of NO and iNOS production. Gate M1 were used to detect NO production (A) and Gate M2 were used to detect iNOS production (B) in PBMC from dogs.
(TIF)

S6 Fig Basal quantification of transcription factors T-bet, GATA3, and FoxP3 in lymphocytes from healthy dogs and dogs with leishmaniasis. Quantification of T-bet, GATA3, and FoxP3 transcription factors was performed on PBMCs on PBMCs from healthy dogs (Control group N = 5) and dogs with leishmaniasis (Infected group N = 18). T-bet (A), GATA3 (B), and FoxP3 (C). Data are expressed as the median and interquartile range (25 and 75). Symbols represent individual data for each animal. Asterisks indicate significant differences (Mann-Whitney test p < 0.05).
(TIF)

S7 Fig Histogram representative of the flow cytometric analysis of the labeling of T-Bet, GATA3 and FoxP3 transcription factors. Gate M1 were used to detect CD3+ lymphocytes (A), Gate M2 to detect CD3+lymphocytes expressing T-bet (B), Gate M3 to detect CD3+lymphocytes expressing GATA3 (C) and Gate M4 to detect CD3+lymphocytes expressing FoxP3(D).
(TIF)

S8 Fig Basal quantification of cytokines in PBMC culture supernatants from healthy dogs and dogs with leishmaniasis. The production of cytokines IL-1-β (A), IL-6 (B), TNFα (C), IFN-y (D), IL-4 (E), IL-10 (F), and TGF-β (G) were measured using capture ELISA. The evaluation was performed on the PBMC culture supernatants from healthy dogs (Control group, N = 5) and dogs with leishmaniasis (Infected group, N = 18). Data are expressed as the median and interquartile range (25 and 75). Symbols represent individual data for each animal. Asterisks indicate significant differences (Mann-Whitney test, p < 0.05)
(TIF)

S9 Fig Basal relative gene expression of TRAF6 in PBMCs from healthy dogs and dogs with leishmaniasis. The relative expression of TRAF6 was analyzed using qPCR. Data are expressed as the median and interquartile range (25 and 75). Symbols represent individual data for each animal. Asterisks indicate significant differences (Mann-Whitney test, p < 0.05)
(TIF)

S10 Fig Histogram representative of the flow cytometric analysis of the labeling of NO production. Gate M1 were used to detect NO production (A) and Gate M2 were used to detect NO production after IL-1β blockade (B) in PBMC from dogs with leishmaniasis.
(JPG)

S1 Table Clinical signs, serological and molecular diagnosis of dogs with leishmaniasis (Infected group) and healthy dogs (Control group). (DOCX)

S2 Table Red blood cell parameters. (DOCX)

S3 Table White blood cells and platelet counts. (DOCX)

S4 Table Sera biochemical profile. (DOCX)

## References

[1] Alvar J, Cañavate C, Molina R, Moreno J, Nieto J. Canine leishmaniasis. Adv Parasitol. 2004;57: 1–88. doi: 10.1016/S0065-308X(04)57001-X 15504537

[2] World Health Organization. Leishmaniasis. No Title. [cited 19 Dec 2022]. Available: https://www.who.int/news-room/fact-sheets/detail/leishmaniasis

[3] Moreno J, Alvar J. Canine leishmaniasis: epidemiological risk and the experimental model. 2002;18: 399–405. 10.1016/s1471-4922(02)02347-4 12377257

[4] Coura-Vital W, Marques MJ, Veloso VM, Roatt BM, Aguiar-Soares RD de O, Reis LES, et al. Prevalence and Factors Associated with Leishmania infantum Infection of Dogs from an Urban Area of Brazil as Identified by Molecular Methods. Boelaert M, editor. PLoS Negl Trop Dis. 2011;5: e1291. doi: 10.1371/journal.pntd.0001291 21858243 PMC3156685

[5] Nunes CM, Pires MM, da Silva KM, Assis FD, Filho JG, Perri SHV. Relationship between dog culling and incidence of human visceral leishmaniasis in an endemic area. Vet Parasitol. 2010;170: 131–133. doi: 10.1016/j.vetpar.2010.01.044 20181428

[6] Pinelli E, Wagenaar J, Bernadina W, Pinelli E, Killick-kendrick R, Wagenaar J, et al. Cellular and Humoral Immune Responses in Dogs Experimentally and Naturally Infected with Leishmania infantum. Infect Immun. 1994;62: 229–235(7). doi: 10.1128/iai.62.1.229-235.1994 8262632 PMC186091

[7] Santos-Gomes GM, Rosa R, Leandro C, Cortes S, Romão P, Silveira H. Cytokine expression during the outcome of canine experimental infection by Leishmania infantum. Vet Immunol Immunopathol. 2002;88: 21–30. doi: 10.1016/s0165-2427(02)00134-4 12088641

[8] Panaro MA, Acquafredda A, Lisi S, Lofrumento DD, Mitolo V, Sisto M, et al. Nitric oxide production by macrophages of dogs vaccinated with killed Leishmania infantum promastigotes. Comp Immunol Microbiol Infect Dis. 2001;24: 187–95. Available: http://www.ncbi.nlm.nih.gov/pubmed/11440191 doi: 10.1016/s0147-9571(00)00026-6 11440191

[9] CABRAL M, O’GRADY J, ALEXANDER J. Demonstration of Leishmania specific cell mediated and humoral immunity in asymptomatic dogs. Parasite Immunol. 1992;14: 531–539. doi: 10.1111/j.1365-3024.1992.tb00026.x 1437241

[10] Bartee E, McFadden G. Cytokine synergy: An underappreciated contributor to innate anti-viral immunity. Cytokine. 2013;63: 237–240. doi: 10.1016/j.cyto.2013.04.036 23693158 PMC3748162

[11] Geraci NS, Tan JC, McDowell MA. Characterization of microRNA expression profiles in Leishmania -infected human phagocytes. Parasite Immunol. 2015;37: 43–51. doi: 10.1111/pim.12156 25376316 PMC4287219

[12] Colineau L, Lambertz U, Fornes O, Wasserman WW, Reiner NE. c-Myc is a novel Leishmania virulence factor by proxy that targets the host miRNA system and is essential for survival in human macrophages. J Biol Chem. 2018;293: 12805–12819. doi: 10.1074/jbc.RA118.002462 29934305 PMC6102154

[13] Ghosh J, Bose M, Roy S, Bhattacharyya SN. Leishmania donovani Targets Dicer1 to Downregulate miR-122, Lower Serum Cholesterol, and Facilitate Murine Liver Infection. Cell Host Microbe. 2013;13: 277–288. doi: 10.1016/j.chom.2013.02.005 23498953 PMC3605572

[14] Melo LM, Bragato JP, Venturin GL, Rebech GT, Costa SF, Garcia LE, et al. Induction of miR 21 impairs the anti-Leishmania response through inhibition of IL-12 in canine splenic leukocytes. Satoskar AR, editor. PLoS One. 2019;14: e0226192. doi: 10.1371/journal.pone.0226192 31825987 PMC6905561

[15] Bragato JP, Melo LM, Venturin GL, Rebech GT, Garcia LE, Lopes FL, et al. Relationship of peripheral blood mononuclear cells miRNA expression and parasitic load in canine visceral leishmaniasis. Afrin F, editor. PLoS One. 2018;13: e0206876. doi: 10.1371/journal.pone.0206876 30517108 PMC6281177

[16] Suzuki HI, Yamagata K, Sugimoto K, Iwamoto T, Kato S, Miyazono K. Modulation of microRNA processing by p53. Nature. 2009;460: 529–533. doi: 10.1038/nature08199 19626115

[17] Hermeking H. MicroRNAs in the p53 network: micromanagement of tumour suppression. Nat Rev Cancer. 2012;12: 613–626. doi: 10.1038/nrc3318 22898542

[18] Romero-Cordoba SL, Salido-Guadarrama I, Rodriguez-Dorantes M, Hidalgo-Miranda A. miRNA biogenesis: biological impact in the development of cancer. Cancer Biol Ther. 2014;15(11):1444–55. doi: 10.4161/15384047.2014.955442 ; PMCID: PMC4622859. 25482951 PMC4622859

[19] Kozomara A, Birgaoanu M, Griffiths-Jones S. miRBase: from microRNA sequences to function. Nucleic Acids Res. 2019;47: D155–D162. doi: 10.1093/nar/gky1141 30423142 PMC6323917

[20] Dong C, Davis RJ, Flavell RA. MAP Kinases in the Immune Response. Annu Rev Immunol. 2002;20: 55–72. doi: 10.1146/annurev.immunol.20.091301.131133 11861597

[21] Chang C-F, D’Souza WN, Ch’en IL, Pages G, Pouyssegur J, Hedrick SM. Polar Opposites: Erk Direction of CD4 T Cell Subsets. J Immunol. 2012;189: 721–731. doi: 10.4049/jimmunol.1103015 22675204 PMC3392534

[22] Ashutosh, Garg M, Sundar S, Duncan R, Nakhasi HL, Goyal N. Downregulation of Mitogen-Activated Protein Kinase 1 of Leishmania donovani Field Isolates Is Associated with Antimony Resistance. Antimicrob Agents Chemother. 2012;56: 518–525. doi: 10.1128/AAC.00736-11 22064540 PMC3256019

[23] Garg M, Goyal N. MAPK1 of Leishmania donovani Modulates Antimony Susceptibility by Downregulating P-Glycoprotein Efflux Pumps. Antimicrob Agents Chemother. 2015;59: 3853–3863. doi: 10.1128/AAC.04816-14 25870075 PMC4468650

[24] Oliveira LG, Souza-Testasicca MC, Ricotta TNQ, Vago JP, dos Santos LM, Crepaldi F, et al. Temporary Shutdown of ERK1/2 Phosphorylation Is Associated With Activation of Adaptive Immune Cell Responses and Disease Progression During Leishmania amazonensis Infection in BALB/c Mice. Front Immunol. 2022;13. doi: 10.3389/fimmu.2022.762080 35145518 PMC8821891

[25] Croker BA, Kiu H, Nicholson SE. SOCS regulation of the JAK/STAT signalling pathway. Semin Cell Dev Biol. 2008;19: 414–422. doi: 10.1016/j.semcdb.2008.07.010 18708154 PMC2597703

[26] Bullen DVR, Baldwin TM, Curtis JM, Alexander WS, Handman E. Persistence of Lesions in Suppressor of Cytokine Signaling-1-Deficient Mice Infected with Leishmania major. J Immunol. 2003;170: 4267–4272. doi: 10.4049/jimmunol.170.8.4267 12682261

[27] Knosp CA, Carroll HP, Elliott J, Saunders SP, Nel HJ, Amu S, et al. SOCS2 regulates T helper type 2 differentiation and the generation of type 2 allergic responses. J Exp Med. 2011;208: 1523–1531. doi: 10.1084/jem.20101167 21646394 PMC3135359

[28] Lomaga MA, Yeh W-C, Sarosi I, Duncan GS, Furlonger C, Ho A, et al. TRAF6 deficiency results in osteopetrosis and defective interleukin-1, CD40, and LPS signaling. Genes Dev. 1999;13: 1015–1024. doi: 10.1101/gad.13.8.1015 10215628 PMC316636

[29] Naito A, Azuma S, Tanaka S, Miyazaki T, Takaki S, Takatsu K, et al. Severe osteopetrosis, defective interleukin-1 signalling and lymph node organogenesis in TRAF6 -deficient mice. Genes to Cells. 1999;4: 353–362. doi: 10.1046/j.1365-2443.1999.00265.x 10421844

[30] Deng L, Wang C, Spencer E, Yang L, Braun A, You J, et al. Activation of the IκB Kinase Complex by TRAF6 Requires a Dimeric Ubiquitin-Conjugating Enzyme Complex and a Unique Polyubiquitin Chain. Cell. 2000;103: 351–361. doi: 10.1016/S0092-8674(00)00126-4 11057907

[31] Wang C, Deng L, Hong M, Akkaraju GR, Inoue J, Chen ZJ. TAK1 is a ubiquitin-dependent kinase of MKK and IKK. Nature. 2001;412: 346–351. doi: 10.1038/35085597 11460167

[32] Kar S, Ukil A, Das PK. Cystatin cures visceral leishmaniasis by NF-κB-mediated proinflammatory response through co-ordination of TLR/MyD88 signaling with p105-Tpl2-ERK pathway. Eur J Immunol. 2011;41: 116–127. doi: 10.1002/eji.201040533 21182083

[33] Srivastav S, Saha A, Barua J, Ukil A, Das PK. IRAK-M regulates the inhibition of TLR-mediated macrophage immune response during late in vitro Leishmania donovani infection. Eur J Immunol. 2015;45: 2787–2797. doi: 10.1002/eji.201445336 26140693

[34] Tian H, Liu C, Zou X, Wu W, Zhang C, Yuan D. MiRNA-194 Regulates Palmitic Acid-Induced Toll-Like Receptor 4 Inflammatory Responses in THP-1 Cells. Nutrients. 2015;7: 3483–3496. doi: 10.3390/nu7053483 25984739 PMC4446763

[35] Kong L, Sun M, Jiang Z, Li L, Lu B. MicroRNA-194 Inhibits Lipopolysaccharide-Induced Inflammatory Response in Nucleus Pulposus Cells of the Intervertebral Disc by Targeting TNF Receptor-Associated Factor 6 (TRAF6). Med Sci Monit. 2018;24: 3056–3067. doi: 10.12659/MSM.907280 29745371 PMC5970547

[36] Lima VMF, Gonçalves ME, Ikeda FA, Luvizotto MCR, Feitosa MM. Anti-leishmania antibodies in cerebrospinal fluid from dogs with visceral leishmaniasis. Braz J Med Biol Res. 2003;36. Available: http://www.scielo.br/pdf/bjmbr/v36n4/4605.pdf doi: 10.1590/s0100-879x2003000400010 12700826

[37] Perosso J, Silva KLO, Ferreira SÍ de S, Avanço SV, dos Santos PSP, Eugênio F de R, et al. Alteration of sFAS and sFAS ligand expression during canine visceral leishmaniosis. Vet Parasitol. 2014;205: 417–423. doi: 10.1016/j.vetpar.2014.09.006 25260330

[38] Sanches L da C, Martini CC de, Nakamura AA, Santiago MEB, Dolabela de Lima B, Lima VMF de. Natural canine infection by Leishmania infantum and Leishmania amazonensis and their implications for disease control. Rev Bras Parasitol Veterinária. 2016;25: 465–469. doi: 10.1590/S1984-29612016071 27925065

[39] Livak KJ, Schmittgen TD. Analysis of Relative Gene Expression Data Using Real-Time Quantitative PCR and the 2−ΔΔCT Method. Methods. 2001;25: 402–408. doi: 10.1006/meth.2001.1262 11846609

[40] Sambrook J, Fritsch EF, Maniatis T. Molecular cloning: a laboratory manual. 2nd ed. Cold Spring Harbor Laboratory, editor. New York: Cold Spring Harbor Laboratory; 1989.

[41] El Tai NO, Osman OF, El Fari M, Presber W, Schönian G. Genetic heterogeneity of ribosomal internal transcribed spacer in clinical samples of Leishmania donovani spotted on filter paper as revealed by single-strand conformation polymorphisms and sequencing. Trans R Soc Trop Med Hyg. 2000;94: 575–579. doi: 10.1016/s0035-9203(00)90093-2 11132393

[42] Di Giorgio C, Ridoux O, Delmas F, Azas N, Gasquet M, Timon-David P. Flow Cytometric Detection of LeishmaniaParasites in Human Monocyte-Derived Macrophages: Application to Antileishmanial-Drug Testing. Antimicrob Agents Chemother. 2000;44: 3074–3078. doi: 10.1128/AAC.44.11.3074-3078.2000 11036025 PMC101605

[43] Pfaffl MW. A new mathematical model for relative quantification in real-time RT-PCR. Nucleic Acids Res. 2001;29: e45. doi: 10.1093/nar/29.9.e45 11328886 PMC55695

[44] Peters IR, Peeters D, Helps CR, Day MJ. Development and application of multiple internal reference (housekeeper) gene assays for accurate normalisation of canine gene expression studies. Vet Immunol Immunopathol. 2007;117: 55–66. doi: 10.1016/j.vetimm.2007.01.011 17346803

[45] Solano-Gallego L, Koutinas A, Miró G, Cardoso L, Pennisi MG, Ferrer L, et al. Directions for the diagnosis, clinical staging, treatment and prevention of canine leishmaniosis. Veterinary Parasitology. 2009. pp. 1–18. doi: 10.1016/j.vetpar.2009.05.022 19559536

[46] Torrecilha RBP, Utsunomiya YT, Bosco AM, Almeida BF, Pereira PP, Narciso LG, et al. Correlations between peripheral parasite load and common clinical and laboratory alterations in dogs with visceral leishmaniasis. Prev Vet Med. 2016;132: 83–87. doi: 10.1016/j.prevetmed.2016.08.006 27664450

[47] Nascimento MSL, Albuquerque TDR, Nascimento AFS, Caldas IS, Do-Valle-Matta MA, Souto JT, et al. Impairment of Interleukin-17A Expression in Canine Visceral Leishmaniosis is Correlated with Reduced Interferon-γ and Inducible Nitric Oxide Synthase Expression. J Comp Pathol. 2015;153: 197–205. doi: 10.1016/j.jcpa.2015.10.174 26590047

[48] Zafra R, Jaber JR, Pérez-Écija RA, Barragán A, Martínez-Moreno A, Pérez J. High iNOS expression in macrophages in canine leishmaniasis is associated with low intracellular parasite burden. Vet Immunol Immunopathol. 2008;123: 353–359. doi: 10.1016/j.vetimm.2008.02.022 18406470

[49] Murray HW, Cartelli DM. Killing of intracellular Leishmania donovani by human mononuclear phagocytes. Evidence for oxygen-dependent and -independent leishmanicidal activity. J Clin Invest. 1983;72: 32–44. doi: 10.1172/jci110972 6308049 PMC1129158

[50] Panaro MA, Fasanella A, Lisi S, Mitolo V, Andriola A, Brandonisio O. Evaluation of Nitric Oxide Production by Leishmania Infantum-infected Dog Macrophages. Immunopharmacol Immunotoxicol. 1998;20: 147–158. doi: 10.3109/08923979809034814 9543705

[51] Kaye PM, Svensson M, Ato M, Maroof A, Polley R, Stager S, et al. The immunopathology of experimental visceral leishmaniasis. Immunol Rev. 2004;201: 239–253. doi: 10.1111/j.0105-2896.2004.00188.x 15361245

[52] Boggiatto PM, Ramer-Tait AE, Metz K, Kramer EE, Gibson-Corley K, Mullin K, et al. Immunologic indicators of clinical progression during canine Leishmania infantum infection. Clin Vaccine Immunol. 2010;17: 267–273. doi: 10.1128/CVI.00456-09 20032217 PMC2815526

[53] Esch KJ, Juelsgaard R, Martinez PA, Jones DE, Petersen CA. Programmed death 1-mediated T cell exhaustion during visceral leishmaniasis impairs phagocyte function. J Immunol. 2013 Dec 1;191(11):5542–50. doi: 10.4049/jimmunol.1301810 Epub 2013 Oct 23. ; PMCID: PMC3896087 24154626 PMC3896087

[54] Menezes-Souza D, Corrêa-Oliveira R, Guerra-Sá R, Giunchetti RC, Teixeira-Carvalho A, Martins-Filho OA, et al. Cytokine and transcription factor profiles in the skin of dogs naturally infected by Leishmania (Leishmania) chagasi presenting distinct cutaneous parasite density and clinical status. Vet Parasitol. 2011;177: 39–49. doi: 10.1016/j.vetpar.2010.11.025 21163578

[55] Figueiredo MM, Deoti B, Amorim IF, Pinto AJW, Moraes A, Carvalho CS, et al. Expression of Regulatory T Cells in Jejunum, Colon, and Cervical and Mesenteric Lymph Nodes of Dogs Naturally Infected with Leishmania infantum. Appleton JA, editor. Infect Immun. 2014;82: 3704–3712. doi: 10.1128/IAI.01862-14 24935975 PMC4187817

[56] Hosein S, Rodríguez-Cortés A, Blake DP, Allenspach K, Alberola J, Solano-Gallego L. Transcription of Toll-Like Receptors 2, 3, 4 and 9, FoxP3 and Th17 Cytokines in a Susceptible Experimental Model of Canine Leishmania infantum Infection. Traub-Csekö YM, editor. PLoS One. 2015;10: e0140325. doi: 10.1371/journal.pone.0140325 26465878 PMC4605763

[57] Mullen AC. Role of T-bet in Commitment of TH1 Cells Before IL-12-Dependent Selection. Science (80-). 2001;292: 1907–1910. doi: 10.1126/science.1059835 11397944

[58] Pai S-Y, Truitt ML, Ho I-C. GATA-3 deficiency abrogates the development and maintenance of T helper type 2 cells. Proc Natl Acad Sci. 2004;101: 1993–1998. doi: 10.1073/pnas.0308697100 14769923 PMC357040

[59] Nylén S, Gautam S. Immunological perspectives of leishmaniasis. J Glob Infect Dis. 2010;2: 135. doi: 10.4103/0974-777X.62876 20606969 PMC2889653

[60] Lima-Junior DS, Costa DL, Carregaro V, Cunha LD, Silva ALN, Mineo TWP, et al. Inflammasome-derived IL-1β production induces nitric oxide–mediated resistance to Leishmania. Nat Med. 2013;19: 909–915. doi: 10.1038/nm.3221 23749230

[61] Alves CF, de Amorim IFG, Moura EP, Ribeiro RR, Alves CF, Michalick MS, et al. Expression of IFN-γ, TNF-α, IL-10 and TGF-β in lymph nodes associates with parasite load and clinical form of disease in dogs naturally infected with Leishmania (Leishmania) chagasi. Vet Immunol Immunopathol. 2009;128: 349–358. doi: 10.1016/j.vetimm.2008.11.020 19124159

[62] Wang K, Lai C, Gu H, Zhao L, Xia M, Yang P, et al. miR-194 Inhibits Innate Antiviral Immunity by Targeting FGF2 in Influenza H1N1 Virus Infection. Front Microbiol. 2017;8. doi: 10.3389/fmicb.2017.02187 29163456 PMC5674008

[63] Vouldoukis I, Bécherel P-A, Riveros-Moreno V, Arock M, Da Silva O, Debré P, et al. Interleukin-10 and interleukin-4 inhibit intracellular killing ofLeishmania infantum andLeishmania major by human macrophages by decreasing nitric oxide generation. Eur J Immunol. 1997;27: 860–865. doi: 10.1002/eji.1830270409 9130636

[64] van der Veen RC. Nitric oxide and T helper cell immunity. Int Immunopharmacol. 2001;1: 1491–1500. doi: 10.1016/s1567-5769(01)00093-5 11515814

[65] Thwe PM, Amiel E. The role of nitric oxide in metabolic regulation of Dendritic cell immune function. Cancer Lett. 2018;412: 236–242. doi: 10.1016/j.canlet.2017.10.032 29107106 PMC5699934

[66] Kröncke K-D, Fehsel K, Suschek C, Kolb-Bachofen V. Inducible nitric oxide synthase-derived nitric oxide in gene regulation, cell death and cell survival. Int Immunopharmacol. 2001;1: 1407–1420. doi: 10.1016/s1567-5769(01)00087-x 11515808

[67] Wong SH, Santambrogio L, Strominger JL. Caspases and nitric oxide broadly regulate dendritic cell maturation and surface expression of class II MHC proteins. Proc Natl Acad Sci. 2004;101: 17783–17788. doi: 10.1073/pnas.0408229102 15598739 PMC539763

[68] Huang D, Cai DT, Chua RYR, Kemeny DM, Wong SH. Nitric-oxide Synthase 2 Interacts with CD74 and Inhibits Its Cleavage by Caspase during Dendritic Cell Development. J Biol Chem. 2008;283: 1713–1722. doi: 10.1074/jbc.M705998200 18003616

[69] Giordano D, Magaletti DM, Clark EA. Nitric oxide and cGMP protein kinase (cGK) regulate dendritic-cell migration toward the lymph-node–directing chemokine CCL19. Blood. 2006;107: 1537–1545. doi: 10.1182/blood-2005-07-2901 16249377 PMC1895400

[70] Sanchez-Robert E, Altet L, Alberola J, Rodriguez-Cortés A, Ojeda A, López-Fuertes L, et al. Longitudinal analysis of cytokine gene expression and parasite load in PBMC in Leishmania infantum experimentally infected dogs. Vet Immunol Immunopathol. 2008;125: 168–175. doi: 10.1016/j.vetimm.2008.04.010 18514330

[71] Corrêa APFL, Dossi ACS, de Oliveira Vasconcelos R, Munari DP, de Lima VMF. Evaluation of transformation growth factor β1, interleukin-10, and interferon-γ in male symptomatic and asymptomatic dogs naturally infected by Leishmania (Leishmania) chagasi. Vet Parasitol. 2007;143: 267–274. doi: 10.1016/j.vetpar.2006.08.023 16979825

[72] Agarwal V, Bell GW, Nam J-W, Bartel DP. Predicting effective microRNA target sites in mammalian mRNAs. Elife. 2015;4. doi: 10.7554/eLife.05005 26267216 PMC4532895

[73] Wang M, Li Z, Zuo Q. miR-194-5p inhibits LPS-induced astrocytes activation by directly targeting neurexophilin 1. Mol Cell Biochem. 2020;471: 203–213. doi: 10.1007/s11010-020-03780-0 32533463

[74] Chen R, Xie F, Zhao J, Yue B. Suppressed nuclear factor-kappa B alleviates lipopolysaccharide-induced acute lung injury through downregulation of CXCR4 mediated by microRNA-194. Respir Res. 2020;21: 144. doi: 10.1186/s12931-020-01391-3 32522221 PMC7288420

[75] Wan S, Li G, Tu C, Chen W, Wang X, Wang Y, et al. MicroNAR-194-5p hinders the activation of NLRP3 inflammasomes and alleviates neuroinflammation during intracerebral hemorrhage by blocking the interaction between TRAF6 and NLRP3. Brain Res. 2021;1752: 147228. doi: 10.1016/j.brainres.2020.147228 33385377

[76] Fichera LE, Albareda MC, Laucella SA, Postan M. Intracellular Growth of Trypanosoma cruzi in Cardiac Myocytes Is Inhibited by Cytokine-Induced Nitric Oxide Release. Infect Immun. 2004;72: 359–363. doi: 10.1128/IAI.72.1.359-363.2004 14688116 PMC343980

