# Supplementary figures and images for: MicroRNA-194 regulates parasitic load and IL-1β-dependent nitric oxide production in the peripheral blood mononuclear cells of dogs with leishmaniasis
Source: PLoS Negl Trop Dis. 2024 Jan 19;18(1):e0011789. doi: 10.1371/journal.pntd.0011789 (PMC10798644; doi:10.1371/journal.pntd.0011789)

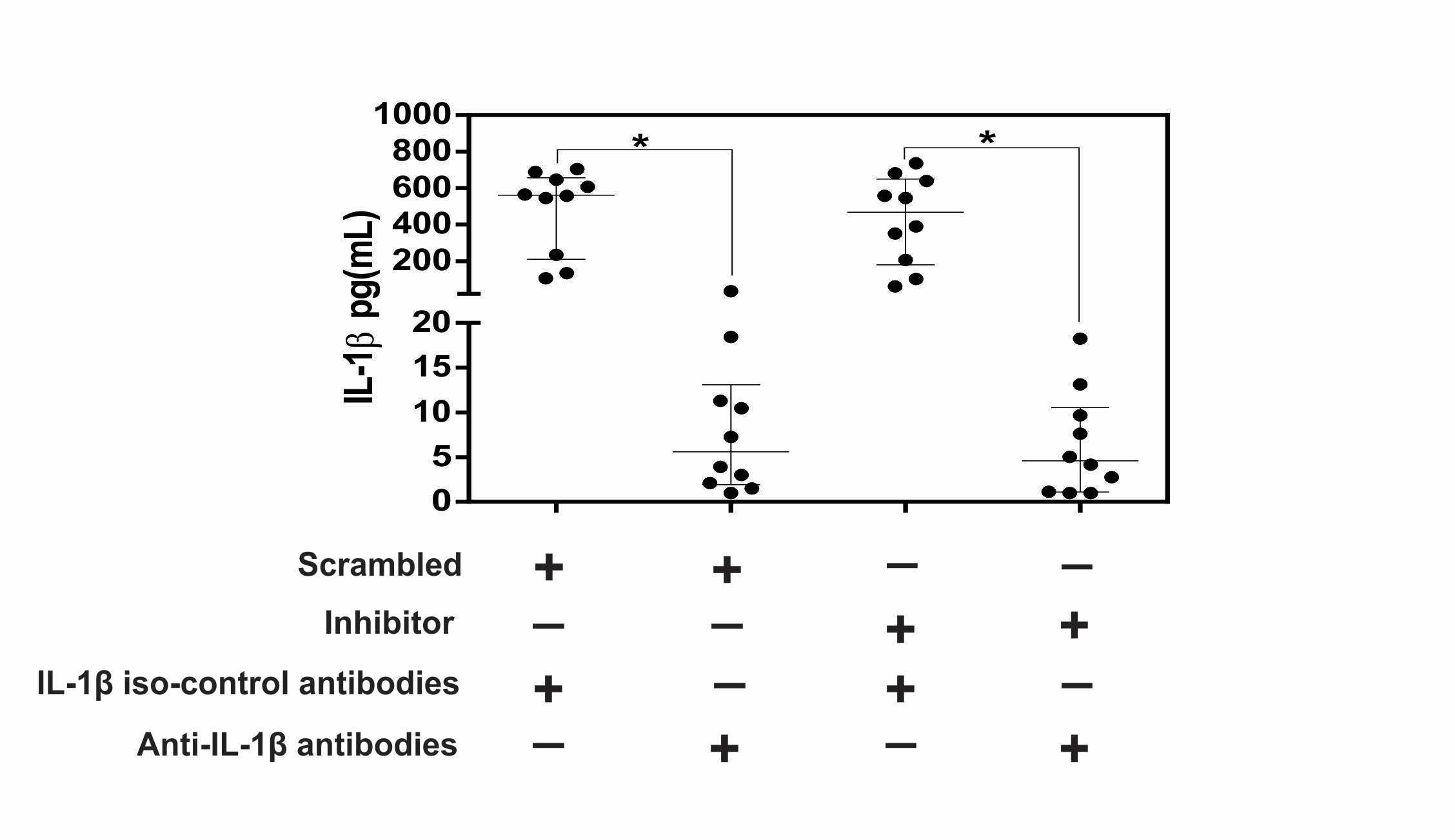

Supplement: S1 Fig — Data are expressed as the median and interquartile range (25 and 75). Symbols represent individual data for each animal. Asterisks indicate significant differences (Friedman’s test followed by Dunn’s test, p < 0.05). (TIF) [file pntd.0011789.s001.tif]

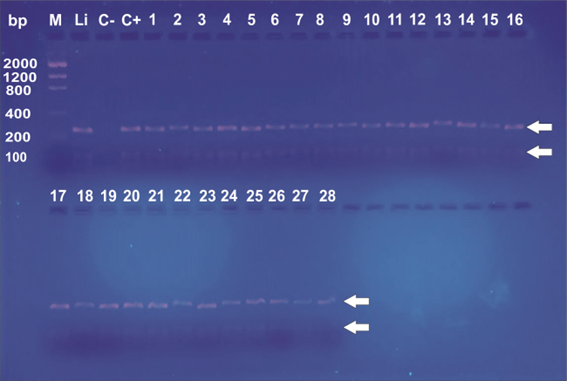

Supplement: S2 Fig — M: molecular marker (100 bp); Li: Leishmania infantum. Samples 1 to 28 showed an identical sampling profile to L. infantum. RFLPs were identified on 3% agarose gels stained with gel red and indicated by an arrow. (TIF) [file pntd.0011789.s002.tif]

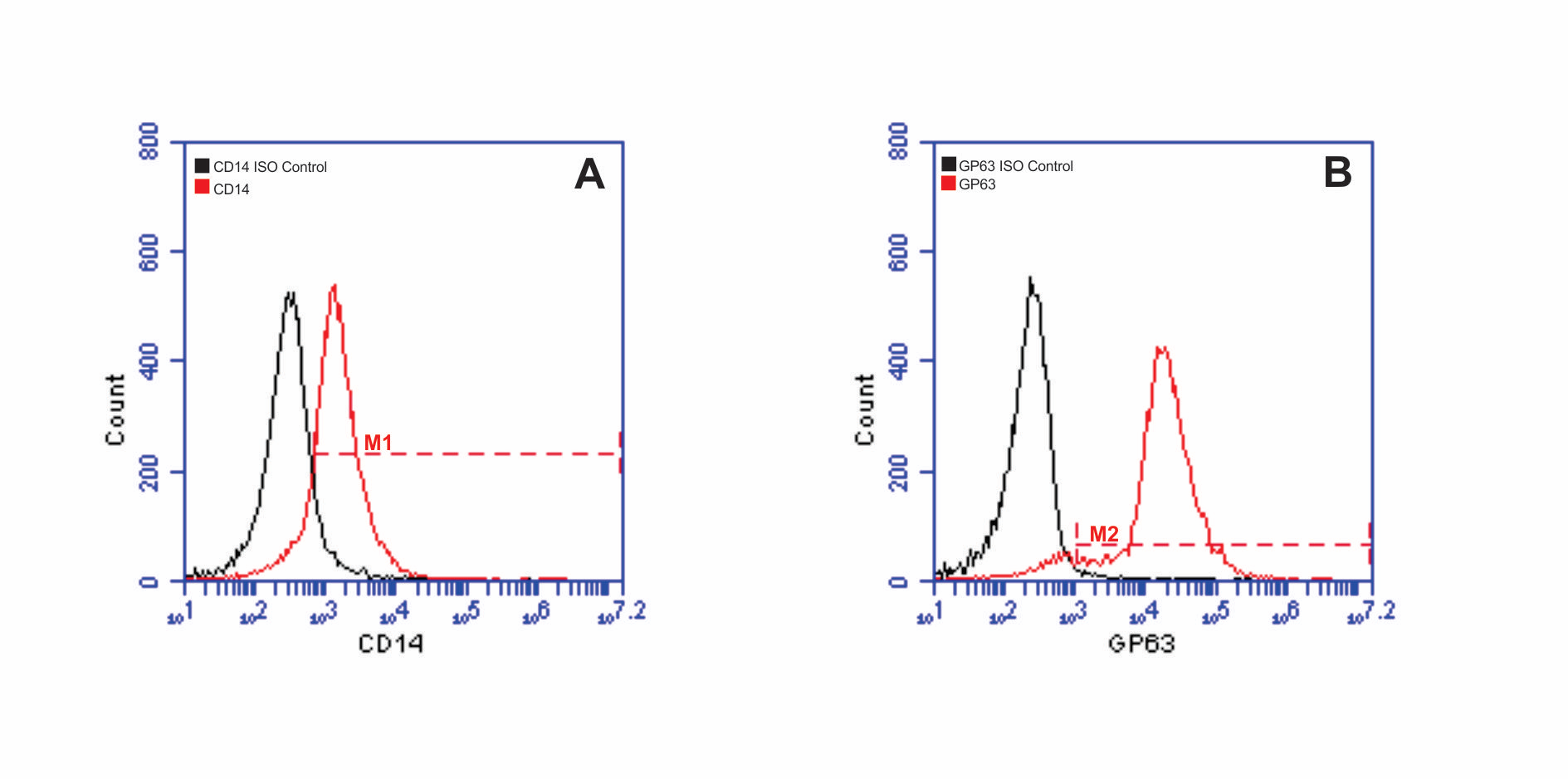

Supplement: S3 Fig — Gate M1 were used to detect CD14+ cells (A) and Gate M2 were used to detect GP63 positive CD14+ cells (B) (TIF) [file pntd.0011789.s003.tif]

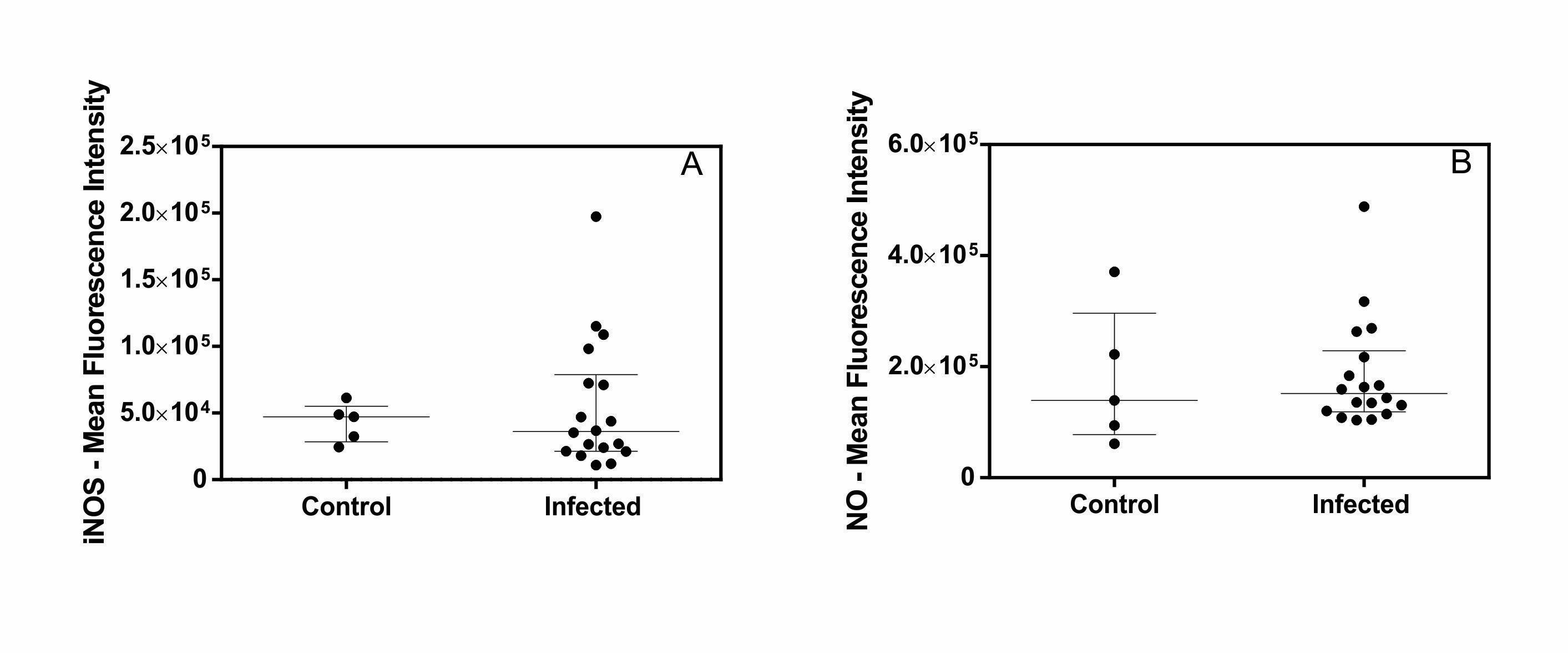

Supplement: S4 Fig — The evaluation of NO production was performed on PBMCs from healthy dogs (Control group, N = 5) (A) and dogs with leishmaniasis (Infected group, N = 18) (B). Data are expressed as the median and interquartile range (25 and 75). Symbols represent individual data for each animal. Asterisks indicate significant differences (Mann-Whitney test, p < 0.05). (TIF) [file pntd.0011789.s004.tif]

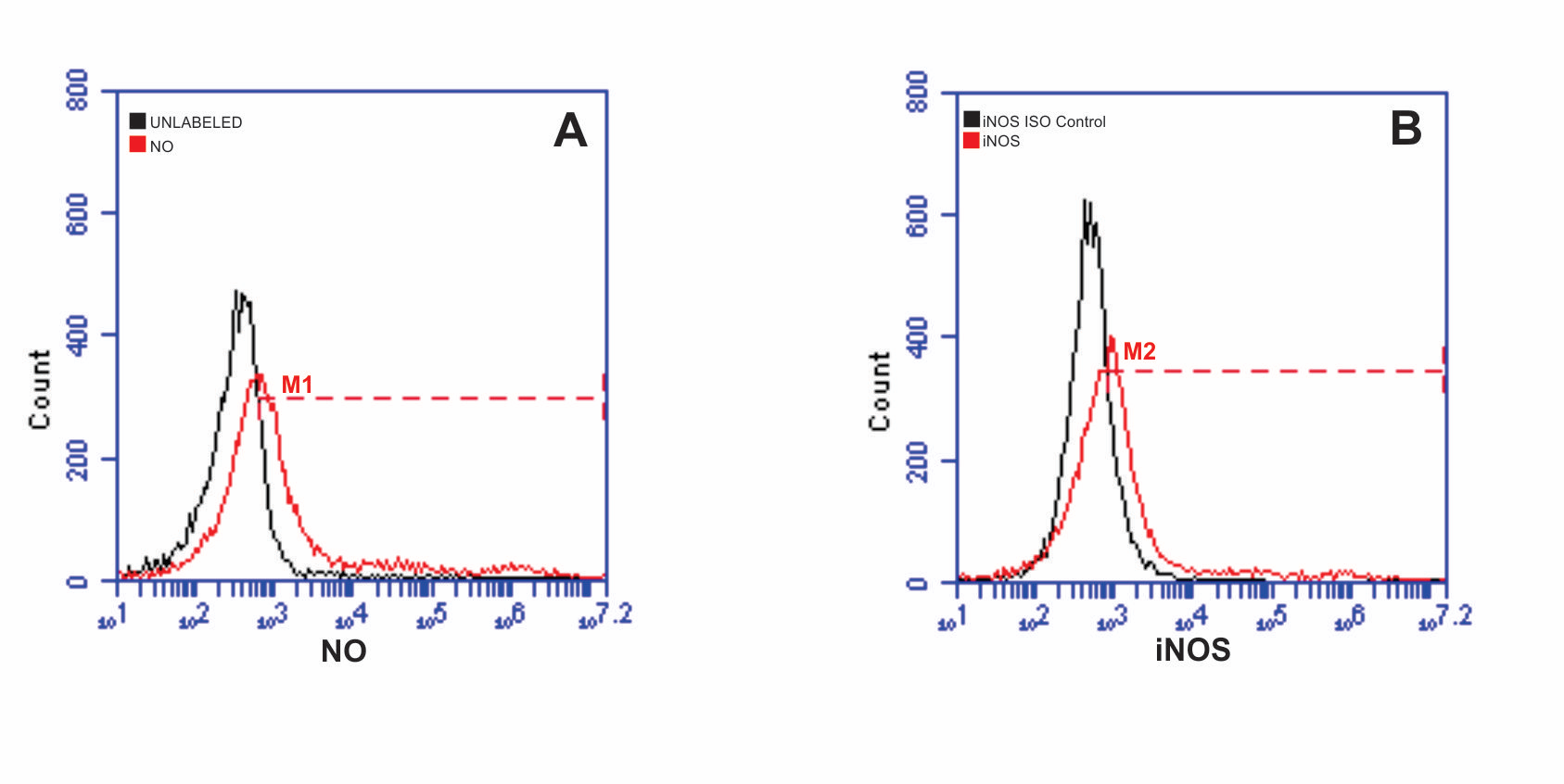

Supplement: S5 Fig — Gate M1 were used to detect NO production (A) and Gate M2 were used to detect iNOS production (B) in PBMC from dogs. (TIF) [file pntd.0011789.s005.tif]

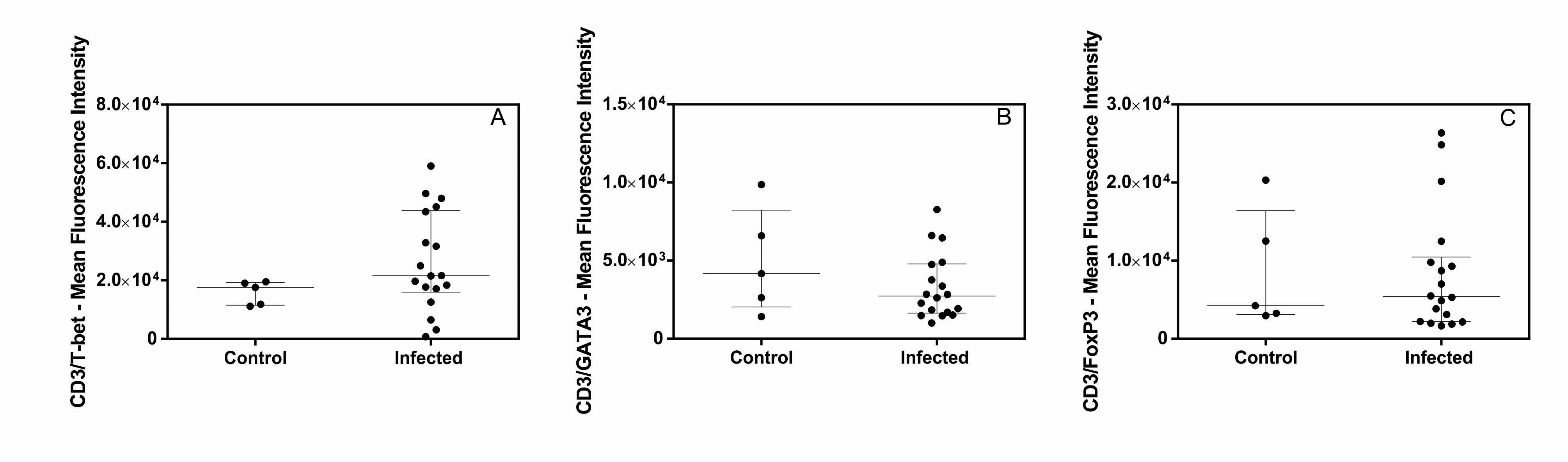

Supplement: S6 Fig — Quantification of T-bet, GATA3, and FoxP3 transcription factors was performed on PBMCs on PBMCs from healthy dogs (Control group N = 5) and dogs with leishmaniasis (Infected group N = 18). T-bet (A), GATA3 (B), and FoxP3 (C). Data are expressed as the median and interquartile range (25 and 75). Symbols represent individual data for each animal. Asterisks indicate significant differences (Mann-Whitney test p < 0.05). (TIF) [file pntd.0011789.s006.tif]

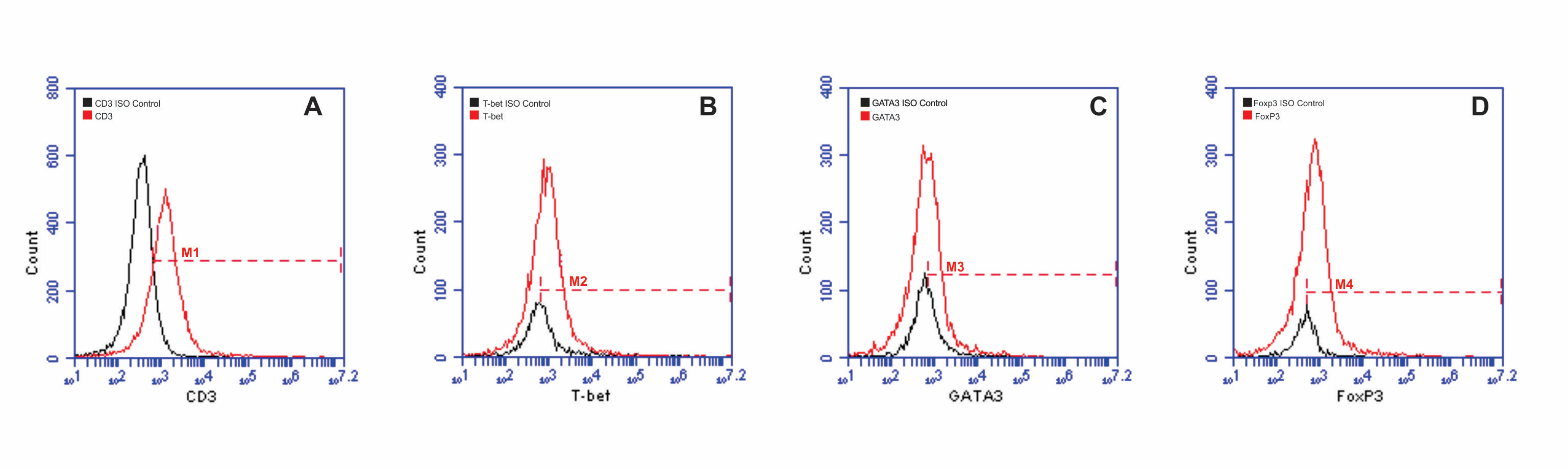

Supplement: S7 Fig — Gate M1 were used to detect CD3+ lymphocytes (A), Gate M2 to detect CD3+lymphocytes expressing T-bet (B), Gate M3 to detect CD3+lymphocytes expressing GATA3 (C) and Gate M4 to detect CD3+lymphocytes expressing FoxP3(D). (TIF) [file pntd.0011789.s007.tif]

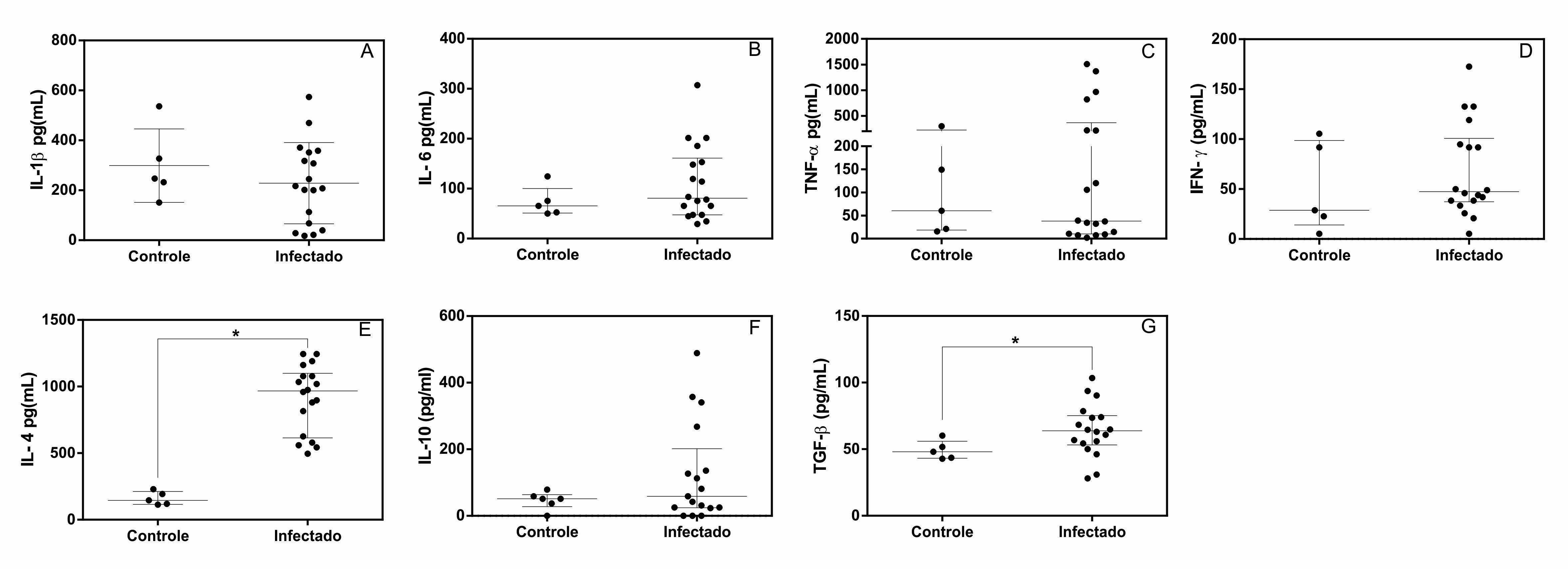

Supplement: S8 Fig — The production of cytokines IL-1-β (A), IL-6 (B), TNFα (C), IFN-y (D), IL-4 (E), IL-10 (F), and TGF-β (G) were measured using capture ELISA. The evaluation was performed on the PBMC culture supernatants from healthy dogs (Control group, N = 5) and dogs with leishmaniasis (Infected group, N = 18). Data are expressed as the median and interquartile range (25 and 75). Symbols represent individual data for each animal. Asterisks indicate significant differences (Mann-Whitney test, p < 0.05) (TIF) [file pntd.0011789.s008.tif]

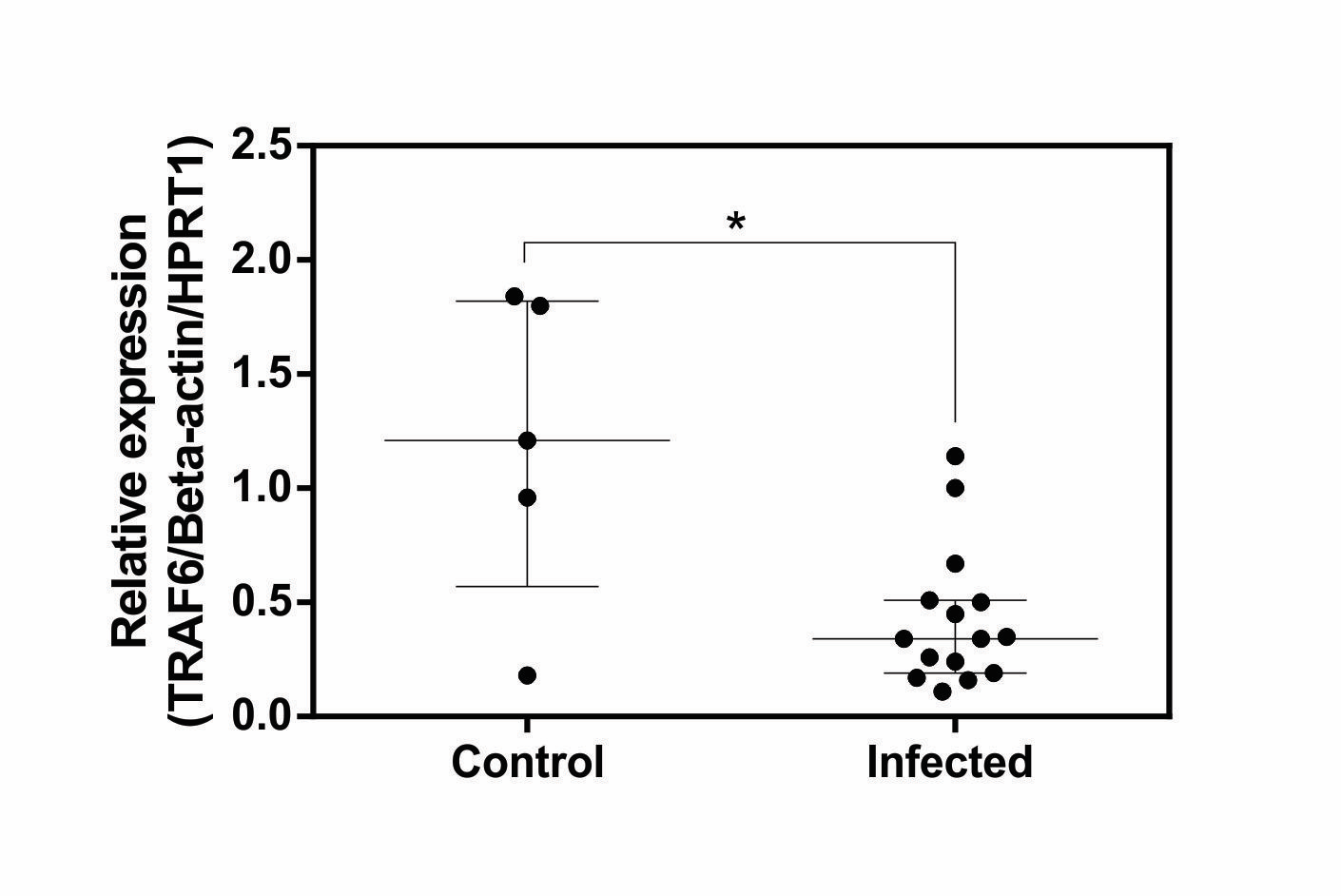

Supplement: S9 Fig — The relative expression of TRAF6 was analyzed using qPCR. Data are expressed as the median and interquartile range (25 and 75). Symbols represent individual data for each animal. Asterisks indicate significant differences (Mann-Whitney test, p < 0.05) (TIF) [file pntd.0011789.s009.tif]

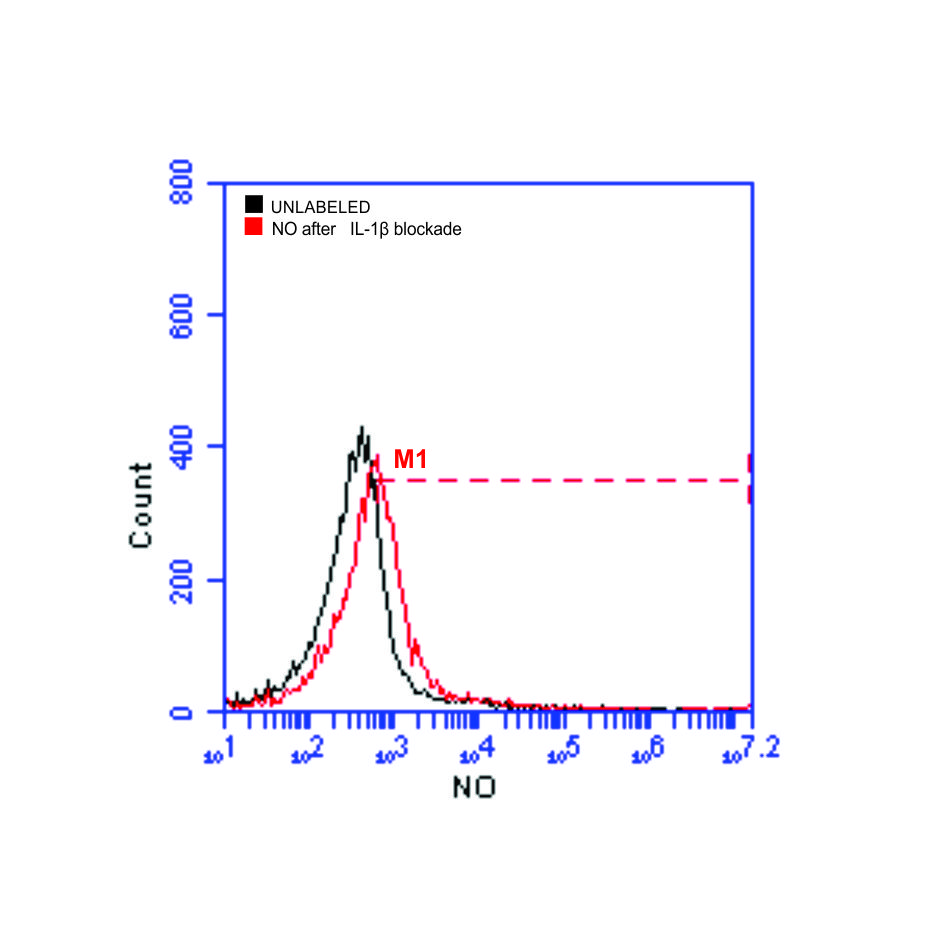

Supplement: S10 Fig — Gate M1 were used to detect NO production (A) and Gate M2 were used to detect NO production after IL-1β blockade (B) in PBMC from dogs with leishmaniasis. (JPG) [file pntd.0011789.s010.jpg]
