# Supplementary material for: MicroRNA-194 regulates parasitic load and IL-1β-dependent nitric oxide production in the peripheral blood mononuclear cells of dogs with leishmaniasis
Source: PLoS Negl Trop Dis. 2024 Jan 19;18(1):e0011789. doi: 10.1371/journal.pntd.0011789 (PMC10798644; doi:10.1371/journal.pntd.0011789)
Supplement: S1 Table — (DOCX) [file pntd.0011789.s011.docx]

| **Dogs** | **Sex** | **Clinical signs** | **Diagnosis** | | |
| --- | --- | --- | --- | --- | --- |
|  |  |  | **DPP** | **PCR** | **ELISA O.D.** |
| Infected 1 | M | Cachexia, seborrhea, skin lesions | + | + | 0.920 |
| Infected 2 | F | Lymphadenopathy, onychogryphosis, alopecia, skin lesions | + | + | 0.978 |
| Infected 3 | M | Lymphadenopathy, onychogryphosis, cachexia, alopecia | + | + | 1.207 |
| Infected 4 | F | Lymphadenopathy, hepatosplenomegaly, onychogryphosis, seborrhea, alopecia, skin lesions | + | + | 1.267 |
| Infected 5 | F | Lymphadenopathy, onychogryphosis, cachexia | + | + | 0.968 |
| Infected 6 | F | Lymphadenopathy, onychogryphosis, cachexia, seborrhea, alopecia, skin lesions | + | + | 0.923 |
| Infected 7 | M | Lymphadenopathy, hepatosplenomegaly, onychogryphosis, skin lesions | + | + | 0.552 |
| Infected 8 | M | Onychogryphosis, cachexia, seborrhea, periocular lesion, skin lesions | + | + | 0.764 |
| Infected 9 | F | Lymphadenopathy, onychogryphosis, cachexia, seborrhea, alopecia, periocular lesion, skin lesions | + | + | 0.649 |
| Infected 10 | M | Lymphadenopathy, hepatosplenomegaly, onychogryphosis, seborrhea, periocular lesion, skin lesions | + | + | 0.912 |
| Infected 11 | M | Lymphadenopathy, hepatosplenomegaly, seborrhea, skin lesions | + | + | 0.295 |
| Infected 12 | M | Lymphadenopathy, onychogryphosis, seborrhea, periocular lesion, skin lesions | + | + | 0.511 |
| Infected 13 | F | Lymphadenopathy, alopecia, skin lesions | + | + | 0.781 |
| Infected 14 | M | Lymphadenopathy, periocular lesion, skin lesions | + | + | 0.986 |
| Infected 15 | M | Cachexia, seborrhea, skin lesions | + | + | 0.687 |
| Infected 16 | F | Cachexia, onychogryphosis, skin lesions | + | + | 0.649 |
| Infected 17 | M | Lymphadenopathy, hepatosplenomegaly, cachexia, skin lesions | + | + | 1.219 |
| Infected 18 | M | Lymphadenopathy, cachexia, skin lesions | + | + | 1.192 |
| Infected 19 | M | Cachexia, periocular injury, skin lesions | + | + | 0.666 |
| Infected 20 | F | Lymphadenopathy, onychogryphosis, cachexia, skin lesions | + | + | 0.570 |
| Infected 21 | M | Lymphadenopathy, onychogryphosis, alopecia, skin lesions | + | + | 0.547 |
| Infected 22 | F | Lymphadenopathy, onychogryphosis, periocular lesion, skin lesions | + | + | 0.814 |
| Infected 23 | F | Lymphadenopathy, periocular lesion, skin lesions | + | + | 0.964 |
| Infected 24 | M | Lymphadenopathy, onychogryphosis, cachexia, skin lesions | + | + | 0.792 |
| Infected 25 | M | Lymphadenopathy, hepatosplenomegaly, onychogryphosis, cachexia, alopecia | + | + | 0.523 |
| Infected 26 | F | Lymphadenopathy, hepatosplenomegaly, onychogryphosis, seborrhea, periocular lesions, skin lesions | + | + | 0.678 |
| Infected 27 | M | Lymphadenopathy, onychogryphosis, alopecia, skin lesions | + | + | 1.328 |
| Infected 28 | F | Hepatosplenomegaly, onychogryphosis, periocular lesion | + | + | 1.209 |
| Control 1 | F | No clinical signs | - | - | 0.112 |
| Control 2 | M | No clinical signs | - | - | 0.070 |
| Control 3 | F | No clinical signs | - | - | 0.094 |
| Control 4 | F | No clinical signs | - | - | 0.045 |
| Control 5 | M | No clinical signs | - | - | 0.016 |

Abbreviations: (DPP) Immunochromatographic test; (PCR) Polymerase Chain Reaction; (O.D.) Optical Density
