# Supplementary material for: MicroRNA-194 regulates parasitic load and IL-1β-dependent nitric oxide production in the peripheral blood mononuclear cells of dogs with leishmaniasis
Source: PLoS Negl Trop Dis. 2024 Jan 19;18(1):e0011789. doi: 10.1371/journal.pntd.0011789 (PMC10798644; doi:10.1371/journal.pntd.0011789)
Supplement: S2 Table — (DOCX) [file pntd.0011789.s012.docx]

| **Dogs** |  | **Red Cells** | **Hemoglobin** | **Hematocrit** | **MCV** | **MCHC** |
| --- | --- | --- | --- | --- | --- | --- |
|  | **Values**  **Reference** | **5.5-8.5**  **x10^12^/L** | **12.0-18.0**  **g/dl** | **37-55**  **%** | **60-77**  **fL** | **32-36**  **%** |
| Infected 1 |  | 4.05 | 7.8 | 25 | 61.73 | 31.20 |
| Infected 2 |  | 3.84 | 9.1 | 25 | 65.10 | 36.40 |
| Infected 3 |  | 4.30 | 9.5 | 27 | 62.79 | 35.19 |
| Infected 4 |  | 4.06 | 10.0 | 28 | 68.97 | 35.71 |
| Infected 5 |  | 2.82 | 6.6 | 20 | 70.92 | 33.00 |
| Infected 6 |  | 4.05 | 10.2 | 30 | 74.07 | 34.00 |
| Infected 7 |  | 3.07 | 7.6 | 23 | 74.92 | 33.04 |
| Infected 8 |  | 4.77 | 10.1 | 29 | 60.80 | 34.83 |
| Infected 9 |  | 5.44 | 11.2 | 34 | 64.34 | 32.00 |
| Infected 10 |  | 4.33 | 9.0 | 28 | 64.67 | 32.14 |
| Infected 11 |  | 5.40 | 11.0 | 35 | 64.34 | 34.29 |
| Infected 12 |  | 5.01 | 11.8 | 36 | 66.18 | 32.78 |
| Infected 13 |  | 5.22 | 11.7 | 35 | 67.05 | 33.43 |
| Infected 14 |  | 5.77 | 14.5 | 44 | 76.26 | 32.95 |
| Infected 15 |  | 5.32 | 11.4 | 34 | 63.91 | 33.53 |
| Infected 16 |  | 4.55 | 10.5 | 32 | 70.33 | 32.81 |
| Infected 17 |  | 4.91 | 11.6 | 34 | 69.25 | 34.12 |
| Infected 18 |  | 5.50 | 14.0 | 39 | 70.91 | 35.90 |
| Infected 19 |  | 5.01 | 11.4 | 33 | 65.87 | 34.55 |
| Infected 20 |  | 5.05 | 11.9 | 34 | 67.33 | 35.00 |
| Infected 21 |  | 5.8 | 11.2 | 36 | 60.24 | 31.85 |
| Infected 22 |  | 4.07 | 11.8 | 37 | 69.42 | 30.98 |
| Infected 23 |  | 3.04 | 7.2 | 20 | 65.79 | 36.00 |
| Infected 24 |  | 4.55 | 10.3 | 30 | 65.93 | 34.33 |
| Infected 25 |  | 2.64 | 6.4 | 18 | 68.18 | 35.56 |
| Infected 26 |  | 5.44 | 10.4 | 34 | 62.50 | 30.59 |
| Infected 27 |  | 4.73 | 10.3 | 30 | 63.42 | 34.33 |
| Infected 28 |  | 3.55 | 7.2 | 22 | 61.97 | 32.73 |
|  | **Mean±SD** | **4.51±0.89^a^** | **10.2±2.3^a^** | **30±6.2^a^** | **66.69±4.21^a^** | **33.69±1.59^a^** |
| Control 1 |  | 6.77 | 17.0 | 52 | 76.81 | 32.69 |
| Control 2 |  | 6.33 | 14.0 | 42 | 66.35 | 33.33 |
| Control 3 |  | 7.07 | 17.0 | 49 | 69.31 | 34.69 |
| Control 4 |  | 7.93 | 18.3 | 53 | 66.83 | 34.53 |
| Control 5 |  | 8.11 | 18.4 | 56 | 69.05 | 32.86 |
|  | **Mean±SD** | **7.24±0.76^b^** | **16.9±1.7^b^** | **50±5.3^b^** | **67.67±4.20^a^** | **33.62±0.93^a^** |

Infected: dogs with leishmaniasis. Control: healthy dogs. RBC: red blood cells, MCV: mean corpuscular, MCHC: mean corpuscular hemoglobin concentration volume. a,b The same letters in the same column indicate no statistical difference using unpaired t-test.
