# Supplementary material for: MicroRNA-194 regulates parasitic load and IL-1β-dependent nitric oxide production in the peripheral blood mononuclear cells of dogs with leishmaniasis
Source: PLoS Negl Trop Dis. 2024 Jan 19;18(1):e0011789. doi: 10.1371/journal.pntd.0011789 (PMC10798644; doi:10.1371/journal.pntd.0011789)
Supplement: S3 Table — (DOCX) [file pntd.0011789.s013.docx]

| **Dogs** |  | | **Neutrophils** | | | **Eosinophils** | | **Basophils** | | | **Monocytes** | | **Lymphocytes** | | **Platelets** | |
| --- | --- | --- | --- | --- | --- | --- | --- | --- | --- | --- | --- | --- | --- | --- | --- | --- |
|  | **Values**  **Reference** | | **3.000-11.500**  **x10^6^/L** | | | **150-1.250 x10^6^/L** | | **Raros**  **x10^6^/L** | | | **150-1.350**  **x10^6^/L** | | **1.000-4.800**  **x10^6^/L** | | **160-400**  **X10^1^** | |
| Infected 1 |  | | 6.831 | | | 198 | | 0 | | | 396 | | 2.475 | | 220 | |
| Infected 2 |  | | 13.783 | | | 358 | | 0 | | | 1.253 | | 2.506 | | 400 | |
| Infected 3 |  | | 4.320 | | | 72 | | 0 | | | 288 | | 1.800 | | 140 | |
| Infected 4 |  | | 6.035 | | | 0 | | 0 | | 425 | | | 2.040 | | 200 | |
| Infected 5 |  | | 9.176 | | | 0 | | 0 | | | 248 | | 2.976 | | 160 | |
| Infected 6 |  | | 3.780 | | | 240 | | 0 | | | 180 | | 1.800 | | 280 | |
| Infected 7 |  | | 4.464 | | | 372 | | 0 | | | 279 | | 4.185 | | 200 | |
| Infected 8 |  | | 9.724 | | | 429 | | 0 | | | 1.144 | | 3.003 | | 180 | |
| Infected 9 |  | | 4.029 | | | 0 | | 0 | | | 316 | | 3.555 | | 160 | |
| Infected 10 |  | | 8.400 | | | 0 | | 0 | | | 120 | | 3.480 | | 160 | |
| Infected 11 |  | | 5.775 | | | 0 | | 0 | | | 154 | | 1.771 | | 200 | |
| Infected 12 |  | | 5.829 | | | 0 | | 0 | | | 174 | | 2.697 | | 300 | |
| Infected 13 |  | | 2.920 | | | 40 | | 0 | | | 400 | | 1.000 | | 160 | |
| Infected 14 |  | | 5.610 | | | 425 | | 0 | | | 185 | | 2.380 | | 200 | |
| Infected 15 |  | | 6.080 | | | 160 | | 0 | | | 160 | | 1.600 | | 400 | |
| Infected 16 |  | | 5.226 | | | 234 | | 0 | | | 178 | | 2.262 | | 280 | |
| Infected 17 |  | | 8.748 | | | 0 | | 0 | | | 324 | | 1.728 | | 380 | |
| Infected 18 |  | | 5.772 | | | 222 | | 0 | | | 444 | | 1.962 | | 400 | |
| Infected 19 |  | | 12.060 | | | 268 | | 0 | | | 154 | | 1.038 | | 300 | |
| Infected 20 |  | | 6.424 | | | 264 | | 0 | | | 188 | | 2.024 | | 280 | |
| Infected 21 |  | | 3.660 | | | 300 | | 0 | | | 180 | | 1.860 | | 180 | |
| Infected 22 |  | | 6.141 | | | 489 | | 0 | | | 178 | | 2.492 | | 220 | |
| Infected 23 |  | | 11.760 | | | 147 | | 0 | | | 735 | | 2.058 | | 180 | |
| Infected 24 |  | | 3.431 | | | 147 | | 0 | | | 194 | | 1.128 | | 160 | |
| Infected 25 |  | | 2.680 | | | 0 | | 0 | | | 179 | | 1.280 | | 200 | |
| Infected 26 |  | | 5.694 | | | 546 | | 0 | | | 156 | | 1.404 | | 160 | |
| Infected 27 |  | | 6.696 | | | 390 | | 0 | | | 620 | | 4.460 | | 180 | |
| Infected 28 |  | | 5.412 | | | 0 | | 0 | | | 198 | | 1.990 | | 160 | |
|  | **Mean±SD** | | **6.445±2.79^a^** | | | **189±174^a^** | | **0±0^a^** | | | **266±148^a^** | | **2.248±0.88^a^** | | **230±82^a^** | |
| Control 1 | |  | | 5.192 | 616 | | 0 | | 352 | | | 2.640 | | 180 | |  |
| Control 2 | |  | | 6.100 | 200 | | 0 | | 150 | | | 3.600 | | 240 | |  |
| Control 3 | |  | | 3.630 | 110 | | 0 | | 155 | | | 1.705 | | 160 | |  |
| Control 4 | |  | | 6.930 | 945 | | 0 | | 155 | | | 2.520 | | 180 | |  |
| Control 5 | |  | | 9.513 | 453 | | 0 | | 151 | | | 4.983 | | 180 | |  |
|  | **Mean±SD** | | **6.273±2.18^a^** | | | **464±335^a^** | | **0±0^a^** | | | **192±89^a^** | | **3.090±1.254^a^** | | **188±30^a^** | |

Infected: dogs with leishmaniasis. Control: healthy dogs. a,b The same letters in the same column indicate no statistical difference using unpaired t-test.
