# Supplementary material for: MicroRNA-194 regulates parasitic load and IL-1β-dependent nitric oxide production in the peripheral blood mononuclear cells of dogs with leishmaniasis
Source: PLoS Negl Trop Dis. 2024 Jan 19;18(1):e0011789. doi: 10.1371/journal.pntd.0011789 (PMC10798644; doi:10.1371/journal.pntd.0011789)
Supplement: S4 Table — (DOCX) [file pntd.0011789.s014.docx]

| **Dogs** |  | **Albumin** | **Globulin** | **T. Protein** | **Creatinine** | **Urea** | **ALT** | **AP** | **GGT** |
| --- | --- | --- | --- | --- | --- | --- | --- | --- | --- |
|  | **Reference values** | **2.6-3.3**  **g/L** | **2.7-4.4**  **g/L** | **5.4-7.1**  **g/L** | **0.5-1.5 mg/dL** | **10.03-50.03 mg/dL** | **21-102 UI/L** | **20-156**  **UI/L** | **1.2-6.4 UI/L** |
| Infected 1 |  | 1.8 | 9.0 | 10.8 | 0.7 | 30 | 37 | 75 | 6.0 |
| Infected 2 |  | 1.6 | 6.4 | 8.0 | 0.8 | 32 | 26 | 120 | 2.0 |
| Infected 3 |  | 1.1 | 5.2 | 6.3 | 1.0 | 60 | 21 | 109 | 1.9 |
| Infected 4 |  | 1.9 | 6.8 | 8.7 | 0.7 | 49 | 92 | 92 | 1.0 |
| Infected 5 |  | 1.0 | 7.4 | 8.4 | 1.5 | 87 | 25 | 135 | 1.0 |
| Infected 6 |  | 2.7 | 8.8 | 11.5 | 0.5 | 28 | 159 | 51 | 1.7 |
| Infected 7 |  | 1.6 | 9.0 | 10.6 | 0.8 | 63 | 20 | 17 | 1.9 |
| Infected 8 |  | 1.9 | 8.9 | 10.8 | 0.5 | 25 | 25 | 107 | 1.1 |
| Infected 9 |  | 1.9 | 7.3 | 9.2 | 0.9 | 44 | 37 | 76 | 1.3 |
| Infected 10 |  | 1.9 | 5.1 | 7.0 | 0.5 | 23 | 27 | 135 | 1.4 |
| Infected 11 |  | 1.7 | 7.1 | 8.8 | 0.7 | 25 | 37 | 35 | 1.3 |
| Infected 12 |  | 1.5 | 9.1 | 10.6 | 1.6 | 70 | 34 | 38 | 2.8 |
| Infected 13 |  | 2.2 | 8.0 | 10.2 | 1.0 | 44 | 22 | 32 | 1.7 |
| Infected 14 |  | 2.3 | 6.2 | 8.5 | 0.7 | 30 | 32 | 76 | 3.2 |
| Infected 15 |  | 2.0 | 8.4 | 10.4 | 0.8 | 42 | 25 | 23 | 2.8 |
| Infected 16 |  | 1.6 | 7.9 | 9.5 | 1.0 | 51 | 37 | 67 | 2.1 |
| Infected 17 |  | 1.8 | 7.6 | 9.4 | 0.9 | 35 | 15 | 57 | 6.0 |
| Infected 18 |  | 2.0 | 4.8 | 7.2 | 0.7 | 32 | 160 | 42 | 4.0 |
| Infected 19 |  | 1.5 | 6.3 | 7.8 | 0.6 | 21 | 45 | 22 | 2.2 |
| Infected 20 |  | 1.7 | 4.5 | 6.2 | 0.5 | 23 | 57 | 141 | 2.1 |
| Infected 21 |  | 1.1 | 5.9 | 7.0 | 0.8 | 33 | 24 | 96 | 1.8 |
| Infected 22 |  | 2.0 | 7.3 | 10.0 | 0.7 | 22 | 40 | 73 | 2.0 |
| Infected 23 |  | 1.0 | 6.0 | 7.0 | 1.1 | 73 | 78 | 151 | 2.0 |
| Infected 24 |  | 1.2 | 5.7 | 6.9 | 1.4 | 56 | 69 | 30 | 3.0 |
| Infected 25 |  | 1.3 | 5.7 | 8.0 | 1.3 | 42 | 57 | 27 | 1.5 |
| Infected 26 |  | 2.3 | 10.0 | 12.3 | 0.7 | 20 | 50 | 51 | 3.0 |
| Infected 27 |  | 1.6 | 10.0 | 11.6 | 1.1 | 36 | 23 | 96 | 2.5 |
| Infected 28 |  | 1.8 | 5.9 | 7.7 | 0.6 | 33 | 66 | 171 | 2.3 |
|  | **Mean±SD** | **1.7±0.4a** | **7.1±1.5^a^** | **7.6±0.8^a^** | **0.8±0.3^a^** | **40±17^a^** | **47±36^a^** | **76±44^a^** | **2.3±1.2^a^** |
| Control 1 |  | 3.3 | 4.3 | 7.1 | 0.9 | 44 | 34 | 35 | 4.1 |
| Control 2 |  | 3.3 | 4.7 | 8.0 | 0.9 | 31 | 30 | 21 | 2,3 |
| Control 3 |  | 3.2 | 4.8 | 9.0 | 0.6 | 28 | 22 | 71 | 4.0 |
| Control 4 |  | 3.2 | 3.8 | 7.0 | 0.9 | 44 | 33 | 69 | 2.7 |
| Control 5 |  | 3.4 | 3.9 | 7.3 | 0.8 | 38 | 36 | 26 | 2.3 |
|  | **Mean±SD** | **3.2±0.8^b^** | **4.3±0.4^b^** | **8.9±1.7^a^** | **0.8±0.1^a^** | **37±7.3^a^** | **31±5.^a^** | **44±23^a^** | **3.0±0.9^a^** |

Infected: dog with leishmaniasis. Control: healthy dogs. ALT: alanine aminotransferase, AST: aspartate aminotransferase, AP: Alkaline phosphatase, GGT: gamma glutamyl transferase. a,b The same letters in the same column indicate no statistical difference using unpaired t-test.
